# Digoxin Suppresses HIV-1 Replication by Altering Viral RNA Processing

**DOI:** 10.1371/journal.ppat.1003241

**Published:** 2013-03-28

**Authors:** Raymond W. Wong, Ahalya Balachandran, Mario A. Ostrowski, Alan Cochrane

**Affiliations:** 1 Department of Laboratory Medicine and Pathobiology, University of Toronto, Toronto, Canada; 2 Department of Molecular Genetics, University of Toronto, Toronto, Canada; 3 Department of Immunology, University of Toronto, Toronto, Canada; Duke University Medical Center, United States of America

## Abstract

To develop new approaches to control HIV-1 replication, we examined the capacity of recently described small molecular modulators of RNA splicing for their effects on viral RNA metabolism. Of the drugs tested, digoxin was found to induce a dramatic inhibition of HIV-1 structural protein synthesis, a response due, in part, to reduced accumulation of the corresponding viral mRNAs. In addition, digoxin altered viral RNA splice site use, resulting in loss of the essential viral factor Rev. Digoxin induced changes in activity of the CLK family of SR protein kinases and modification of several SR proteins, including SRp20 and Tra2β, which could account for the effects observed. Consistent with this hypothesis, overexpression of SRp20 elicited changes in HIV-1 RNA processing similar to those observed with digoxin. Importantly, digoxin was also highly active against clinical strains of HIV-1 *in vitro*, validating this novel approach to treatment of this infection.

## Author Summary

Antiretroviral therapies (ART) for HIV/AIDS are successful in slowing disease progression by inhibiting viral proteins. However, the ability of HIV to adapt to ARTs has given rise to drug-resistant virus strains that now represent ≥16% of newly infected people. This development calls for the generation of new treatment strategies. Since HIV is dependent upon RNA processing under control of the host, we searched for compounds/drugs that inhibit HIV-1 replication at this step. We identified digoxin as a potent inhibitor of HIV-1 replication. The drug inhibited expression of HIV-1 structural proteins and a key factor involved in viral RNA export. This response was accomplished by altering the efficiency and splicing choices in HIV-1 RNA processing. Since this stage of the virus lifecycle is not targeted by current ARTs, the digoxin family of drugs represent a novel class of HIV-1 inhibitors. Since digoxin targets host factors and is already in clinical use, it and potentially the cardiac glycoside family of drugs has the possibility for swift development into a new ART for HIV-1 infection.

## Introduction

Current highly active anti-retroviral therapies (HAARTs) have successfully delayed the progression of HIV-1-infected individuals to AIDS by targeting viral entry and all HIV-1 enzymes [1], [2]. However, the clinical application of ARTs is being affected by the spread of drug resistant viral strains [3], [4], [5]; detection of drug resistant forms of HIV-1 in newly infected patients has increased ∼3-fold from 2000 to 2007 to 16% [6], [7]. To overcome these hurdles, more drugs with better profiles, and especially, novel mechanisms of action, are necessary for continued success in combating HIV-1 [1], [2], [8]. However, the majority of drugs currently undergoing clinical trials target the same enzymes/proteins for which drugs are already available [1], [2], [9], [10]. In addition, the persistence of virus in reservoirs continues to be a challenge with standard HAART.

There are at least 200 host factors required for HIV-1 infection and replication [11], [12], [13]. Efforts to understand the role of these factors in the lifecycle of HIV could aid development of future therapies. Among these are the factors regulating RNA processing. HIV-1 requires a balanced regulation of viral RNA processing to generate >40 mRNAs for synthesis of 15 viral proteins, an effect achieved through alternative splicing of a single 9 kb pre-mRNA transcript (Fig. S1) [14], [15], [16], [17]. HIV-1 RNA processing involves the combinatory use of four 5′ splice sites (splice donors, SD1–4) and eight suboptimal 3′ splice sites (splice acceptors, SA1–7; Fig. S1). Use of 3′ splice sites (ss) is regulated by host trans-acting factors that function in an antagonistic fashion by binding to cis-acting elements adjacent to the 3'ss, either impeding (hnRNPs) or promoting (SR proteins) their use [14], [16], [18], [19], [20]. Three classes of HIV-1 mRNAs result from HIV-1 RNA splicing (Fig. S1): unspliced RNAs (US) encoding Gag or Gagpol proteins, singly spliced RNAs (SS) producing Env, Tat (p14), Vif, Vpr, or Vpu, and multiply spliced RNAs (MS) for synthesis of Rev, Tat (p16), or Nef [16], [17], [21]. Among these, Tat and Rev factors play central roles in HIV-1 replication; Tat activates transcription of all viral RNAs, while Rev transports the incompletely-spliced RNAs (US, SS) to the cytoplasm for translation [14], [22], [23], [24], [25], [26]. Imbalances in RNA processing can dramatically affect viral replication [27], [28], [29]; undersplicing results in the loss of key regulatory proteins such as Tat and Rev (from MS RNA), while oversplicing would reduce incompletely-spliced RNAs (US, SS) encoding viral structural proteins (Gag, Env) and accessory factors (Vif, Vpr, Vpu).

Knowledge of how to manipulate these processes to alter HIV-1 RNA splicing in cells could prove advantageous as a strategy for controlling HIV infection. This hypothesis is supported by studies where modulating SR protein abundance (by overexpression/depletion) caused imbalances in HIV-1 splicing, resulting in gross changes in viral protein synthesis [18], [20], [30], [31]. This hypothesis is also supported by the observation that HIV-1 infection leads to a decrease in overall SR protein/activity which can be reversed by increasing SR protein kinase (SRPK) 2 function [32]. Consistent with these studies, we have successfully suppressed HIV-1 gene expression through modulation of another family of SR protein kinases, the Cdc2-like kinases (CLKs) [33]. While use of small molecular weight (MW) inhibitors of SRPK 1 and 2 have met with limited effect against HIV [32], we recently demonstrated that chlorhexidine (an inhibitor of CLKs 2, 3, and 4) is able to alter HIV-1 RNA processing, leading to inhibition of HIV-1 replication [33]. However, the toxicity of chlorhexidine in peripheral blood mononuclear cell (PBMC) cultures precludes its systemic use. Further supporting the viability of this approach is recent work demonstrating the suppression of HIV-1 RNA splicing using indole derivatives that function by modulating SR protein function [19], [34], [35].

To explore this strategy further, we tested compounds shown to modulate host alternative RNA splicing to identify new inhibitors of HIV-1 replication [36], [37]. We report here that digoxin, a drug widely used in treatment of congestive heart failure [38], [39], is a potent inhibitor of HIV-1 replication. Digoxin treatment drastically reduced HIV-1 gene expression in stably HIV-1 transduced HeLa and SupT1 cell lines and is effective in inhibiting replication of HIV-1 clinical strains in human CD4^+^ PBMCs. Digoxin accomplishes these effects through two mechanisms: inducing oversplicing of HIV-1 RNA, resulting in an alteration in splice site usage of HIV-1 pre-mRNA as well the loss of the key regulatory protein, Rev. Consequently, this response impairs expression of viral structural proteins. Reduced Rev expression leads to HIV-1 incompletely-spliced RNAs (US, SS) being sequestered in the nucleus. Expression of Rev *in trans* led to a partial rescue of HIV-1 structural protein (Gag) synthesis. Coincident with the changes in viral RNA processing, digoxin treatment also induced changes in the modification of a subset of SR proteins (SRp20, Tra2β, SRp55, and SRp75) and the activity of the CLK family of SR protein kinases. Our findings support the hypothesis that HIV-1 RNA processing can be effectively targeted without severe toxicity to the host cell. Since this stage of the virus lifecycle is not targeted by current anti-retroviral therapies (ART) [1], [2], digoxin (and potentially the cardiac glycoside family of drugs) represent a novel class of HIV-1 inhibitors with the potential for rapid development into an ART.

## Results

### Digoxin is a potent inhibitor of HIV-1 gene expression

**Figure 1:**
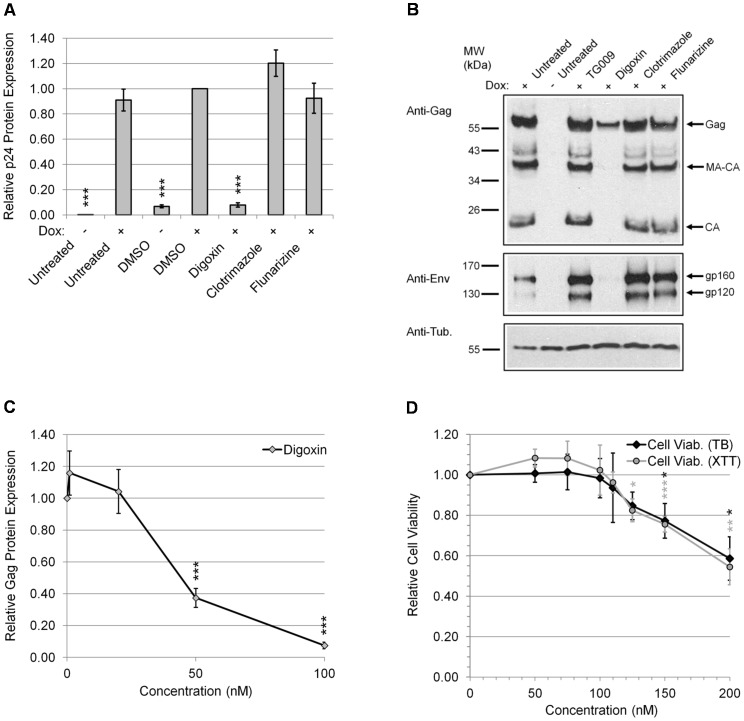
Digoxin suppresses HIV-1 gene expression. HeLa rtTA-HIV-Δ*Mls* cells were treated with indicated drugs for 4 h prior to induction of viral gene expression with (+) and without (−) Dox for ∼20 h. In (A, B), cells were either untreated or treated with 100 nM of digoxin, 10 µM of clotrimazole, 10 µM of flunarizine, or DMSO solvent. Equal concentrations of DMSO were present in each treatment (A–D). In (B), TG009 (an inactive analog of the CLK inhibitor, TG003) served as an additional control with untreated (+) control. (A, C, D) Cell culture supernatants were harvested after drug treatments for analysis of HIV-1 Gag protein expression by p24^CA^ ELISA. Peak Gag expression averaged ∼1200 pg/ml in media harvested from induced cells. Data was averaged from *n*≥12, displayed as a fraction relative to DMSO (+) control, error bars are SEM, and statistical significance (drug treatments vs. DMSO (+) control) indicated by asterisks as described in “Materials & Methods”. (B) Cell lysates were analyzed by western blot of Gag (p55) as well as its processing intermediates (MA-CA, p41; CA, p24) (top) and Env (middle) while tubulin (bottom) served as an internal loading control. Data is representative of ≥4 independent experiments. (C, D) Dose-response curves characterizing digoxin inhibition of HIV-1 gene expression. Cells treated with digoxin (0–100 nM) were evaluated for (C) inhibition of Gag protein expression as described above or (D) effects on cell viability (0–200 nM) by Trypan blue (TB) (black diamonds) and XTT (gray circles). Inhibition data was averaged from *n*≥7 and cell health data was *n*≥6 and displayed as a fraction relative to DMSO (+) control, error bars are SEM, and statistical significance indicated by asterisks as mentioned above.

In our search for novel HIV-1 inhibitors, drugs with the capacity to alter RNA splicing were screened for antiretroviral activity [36], [37]. We used a human cell line stably transduced with a modified X4 HIV-1 (LAI) provirus regulated by a Tet-ON system that requires addition of doxycycline (Dox) for activation of viral gene expression [33], [40], [41]. The effects of drugs on HIV-1 gene expression were monitored by treating HeLa rtTA-HIV-Δ*Mls* cells for 4 hours with drugs prior to induction of virus gene expression by Dox (Fig. 1). After 20 hours, media and cell lysates were harvested for analysis of HIV-1 Gag protein expression by p24^CA^ ELISA (Fig. 1A) or Western blots for Gag and Env (gp120) (Fig. 1B, top and middle, respectively). We observed that digoxin (100 nM) caused a 94% inhibition of HIV-1 Gag protein expression relative to DMSO control (Fig. 1A). In contrast, other drugs shown to affect RNA splicing such as clotrimazole and flunarizine (10 µM) showed no significant effects [36]. Western blot analysis of Gag protein expression in cell lysates of digoxin-treated cells (Fig. 1B, top) confirms a complete loss of the Gag products, capsid (CA) and matrix (MA)-CA, and a marked reduction in Gag protein species relative to controls (untreated and TG009, +). Western blot analysis of Env (Fig. 1B, middle) demonstrated a loss in both gp120 and gp160 proteins to near undetectable levels compared to controls. Upon subsequent analysis of the dose response curve (Fig. 1C), digoxin demonstrated potent inhibition of HIV-1 Gag protein expression with an IC_50_ of ∼45 nM (IC_90_ = 100 nM). Parallel assessment of the cytotoxicity of digoxin treatment on this cell line (Fig. 1D) revealed no significant effects on cell viability at the dose ranges required to inhibit HIV-1 gene expression (50–100 nM) as measured by XTT and Trypan blue (TB) exclusion assays (0–200 nM) (Fig. 1D).

### Digoxin inhibits HIV-1 replication in PBMCs

**Figure 2:**
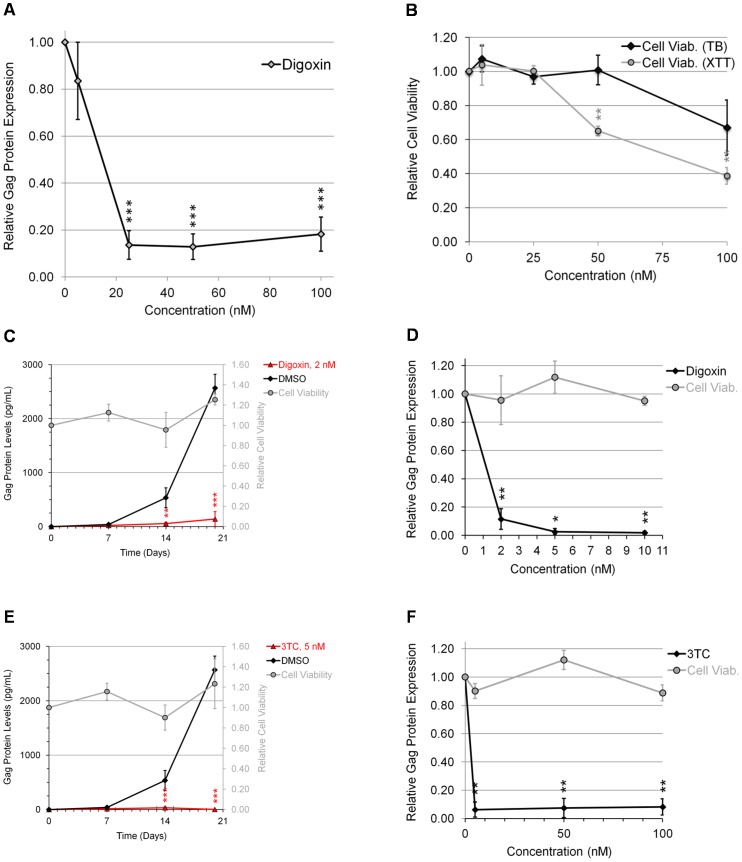
Digoxin inhibits HIV-1 replication in PBMCs. (A–B) PHA/IL-2-activated human PBMCs were infected with R5 BaL strain of HIV-1 at a MOI of 10^−2^. Following infection, cells were incubated in the presence of indicated doses of digoxin or DMSO (control). Equal concentrations of DMSO were present in each treatment. (A) After 8–10 days, media was harvested for analysis of HIV-1 Gag protein expression by p24^CA^ ELISA. Peak levels of Gag expression averaged ∼800 pg/ml at ∼8 days post-infection. ELISA results were averaged from ≥4 experiments and displayed as a fraction relative to DMSO (+) control, error bars are SEM, and statistical significant changes of treatments from DMSO control are indicated by asterisks as detailed in “Materials & Methods”. (B) Cells were assessed following digoxin treatment for cell viability by Trypan blue exclusion (TB, black diamonds) or XTT assay (grey circles) as indicated. Shown is data averaged from ≥5 and ≥3 experiments, respectively, and displayed as a fraction relative to DMSO (+) control, error bars are SEM, and statistical significance indicated by asterisks as mentioned above. (C–F) CD8^+^-depleted PBMCs from chronically HIV-1-infected patients were activated by treatment with anti-CD3 and anti-CD28 antibodies to induce virus growth in cell culture. Cells were treated with either (C, D) digoxin or (E, F) the NRTI, 3TC. Media was harvested at multiple times points to assess virus replication by p24^CA^ ELISA of Gag. Shown (C, E) are the viral growth curves from 20 days of cell culture and (D, F) the dose response effects of the indicated drugs after only 14 days of culture. Effect of drugs on cell viability (Cell Viab., grey circles) was also tested in parallel by XTT assay. Note: cell viability data for (C, E) are shown on the y-axis adjacent to Gag levels while (D, F) is displayed in a similar manner but on the same y-axis as Gag expression. Inhibition and viability results shown (C–F) are derived from assays performed on three different patients, expressed relative to DMSO-treated cells, error bars are SEM, and statistical significance indicated by asterisks as described above.

To validate our findings in a more relevant setting, the ability of digoxin to suppress HIV-1 replication in the context of human CD4^+^ PBMCs was examined. Isolated PBMCs were infected with a R5 BaL strain of HIV-1 in the presence of increasing doses of digoxin and the extent of virus replication was monitored by p24^CA^ ELISA (Fig. 2A). Analysis of the data revealed a profound suppression of HIV-1 replication upon addition of digoxin (IC_90_ = ∼25 nM). Parallel examination of the effect of these treatments on cell viability (Fig. 2B) determined that negative effects were only discernible at doses of ≥50 nM (by XTT assay), above the dose required to strongly suppress HIV-1 replication. In comparison to the stable cell line, analysis of media from PBMC infections at earlier time points (day 3; Fig. S2), representing less cycles of replication, demonstrated significant reduction in HIV-1 replication without significant effects on cell viability (data not shown). As a further test of the efficacy of digoxin in suppressing HIV-1 replication, a similar trial was performed using CD8^+^-depleted PBMCs obtained from treatment-naïve HIV-infected patients. As shown in Fig. 2C and 2E, while Gag accumulated over time in control samples (DMSO), digoxin inhibited HIV-1 replication over the 20 days of the assay to a level comparable to the nucleoside reverse transcriptase inhibitor (NRTI), 3TC (Fig. 2E and 2F). Furthermore, dose response curves (Fig. 2D) demonstrate inhibition of HIV-1 replication at an IC_90_ of 2 nM with no detectable effects on cell viability.

### Digoxin alters HIV-1 RNA processing

**Figure 3:**
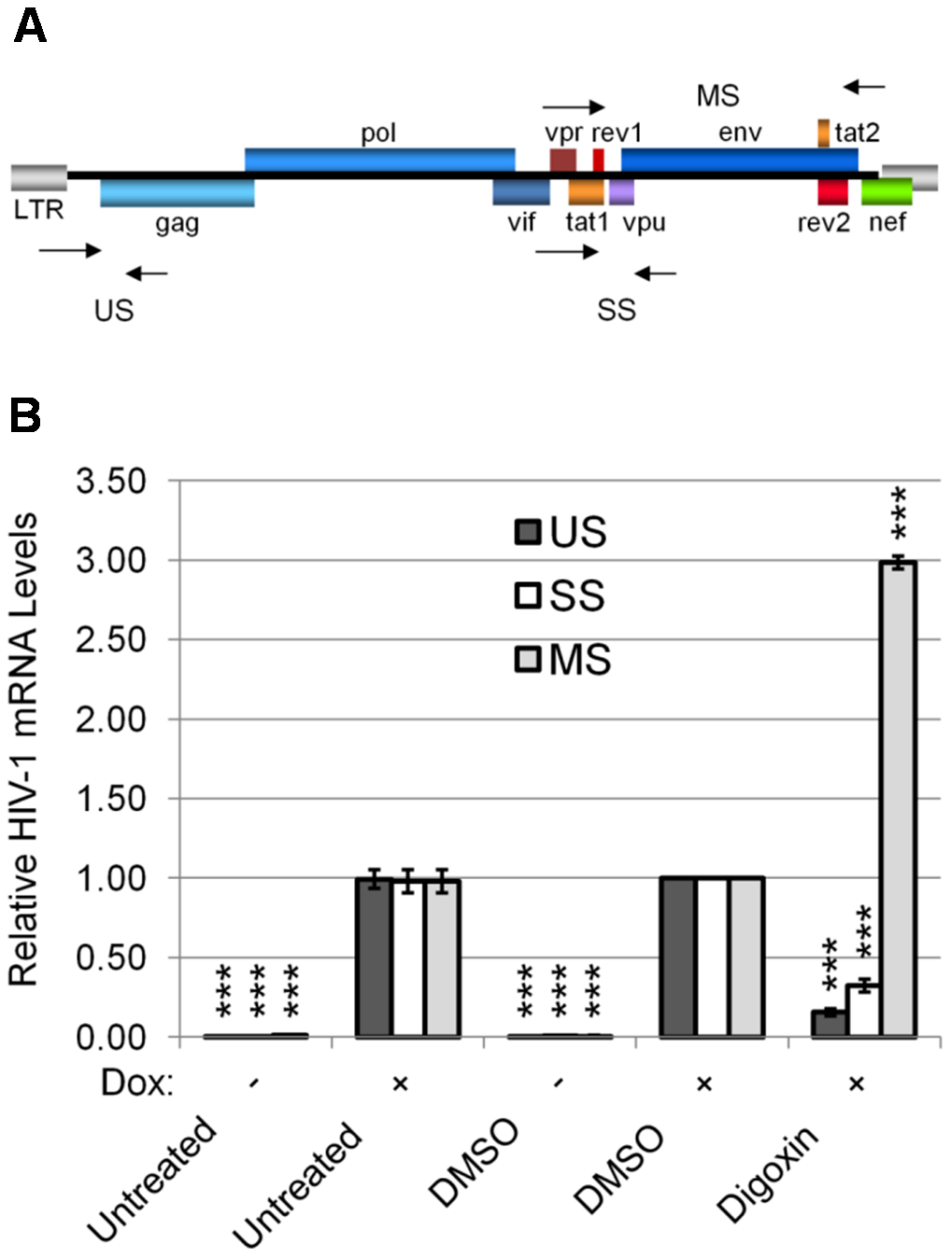
Digoxin alters HIV-1 RNA processing. The effect of digoxin (100 nM) on HIV-1 mRNA levels was assessed by qRT-PCR of cells treated with drug or DMSO as described in Fig. 1. Equal concentrations of DMSO were present in each treatment. (A) Diagram indicating the position of the primers (arrow points) used in qRT-PCR (see Methods). (B) Abundance of HIV-1 unspliced (US, black), singly spliced (SS, white), and multiply spliced (MS, gray) mRNAs are shown relative to DMSO (+) controls. The housekeeping gene, β-actin, served as an internal loading control for the normalization of this data. Dox induced (+) compared to uninduced (−) shows successful activation of HIV-1 gene expression. Shown is data averaged from ≥6 experiments, error bars are SEM, and significant changes of treatments from DMSO (+) control are indicated by asterisk as described in “Materials & Methods”.

To determine the mechanism underlying the response to digoxin, we analyzed its effect on the abundance of all three classes of HIV-1 mRNA by qRT-PCR (Fig. 3). Using the HeLa HeLa rtTA-HIV-Δ*Mls* cell line, digoxin treatment induced an 84% reduction in US mRNA levels (encoding Gag and Gagpol) and a 68% decrease in SS mRNA (encoding Env, p14 Tat, Vpr, Vif, or Vpu). In contrast, digoxin increased MS mRNA (p16 Tat, Rev, Nef) by 300%. The effect of digoxin on HIV-1 RNA abundance was also dose dependent (Fig. S3), in agreement with its effects on the expression of viral structural proteins, Gag and Env (Fig. 1). These results are consistent with digoxin inhibition being due to the induction of viral RNA oversplicing, which is in contrast to the inhibition of splicing induced by indole derivatives [19], [35], [42]. The response to digoxin results in a specific loss of larger, incompletely-spliced mRNA species (encoded by US and SS) that, in turn, reduces the synthesis of proteins necessary for virus assembly. To validate that the response observed was not unique to the HeLa cell line, assays were repeated in 24ST1NLESG cells, a human T cell line (SupT1) chronically infected with a HIV-1 variant (NLE^−^S-G, a pNL4-3-based virus vector) [43]. Assays determined that digoxin also suppressed HIV-1 Gag expression in the SupT1 cell line (Fig. S4C), inducing a similar reduction in abundance of incompletely-spliced viral RNAs (US, SS) and increasing MS RNA accumulation (Fig. S4D) as seen for HeLa rtTA-HIV-Δ*Mls* cells (Fig. 3).

### Digoxin alters the usage of specific HIV-1 pre-mRNA splice sites

**Figure 4:**
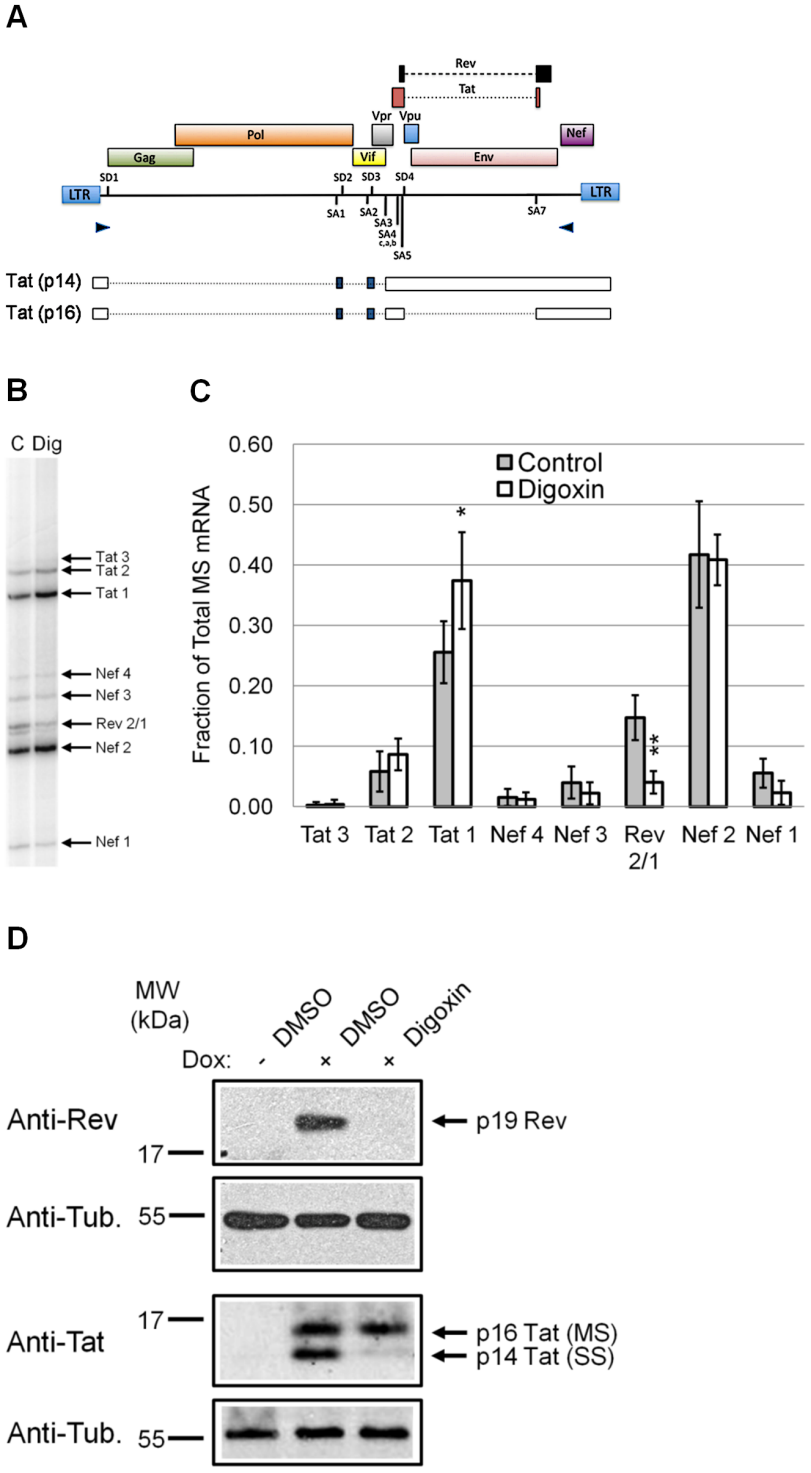
Digoxin alters HIV-1 pre-mRNA splice site usage to suppress Rev expression. Effects of digoxin on HIV-1 splice site use were assayed by RT-PCR of the 2 kb, MS class of HIV-1 mRNA. (A) Diagram of the HIV-1 provirus indicating the splice sites (SD1–4, SA1–7) and position of the primers (arrow points) used to amplify MS mRNA species (see Materials and Methods). Also shown below are the forms of viral RNA used to express the two isoforms of Tat: p14 generated from SS RNA and p16 from MS RNA. Open boxes represent exons, closed boxes are alternative exons, and dashed lines are excised introns. The SS RNA generates a truncated form of Tat (p14) because of the presence of a termination codon immediately 3′ of SD4. (B) A representative RT-PCR gel of the levels of each MS mRNA species (arrows) from cells treated with digoxin (Dig) or control (C) using cDNAs described in Fig. 3. (C) Graph summarizing the effects of digoxin (white) and control treatments (gray) on the level of each MS mRNA species (x-axis) relative to the total HIV-1 MS mRNA (y-axis) and displayed as a fraction of the total MS HIV-1 RNA. Data was averaged from ≥6 experiments, error bars are SEM, and significant changes of treatments from DMSO (+) control are indicated by asterisks. (D) Western blot analysis of HIV-1 regulatory factors Rev (top) and Tat (bottom) from cells treated with digoxin or DMSO as described in Fig. 1. Anti-α-tubulin blots served as an internal loading control for the relative amount of protein lysate in each lane. In each experiment, Dox induced (+) compared to uninduced (−) shows successful activation of HIV-1 protein expression. Protein products (right), specific antibodies detecting them (left), and MW standards (top left) are as indicated. Each immunoblot is a representative of the effects observed from ≥7 independent experiments.

To analyze the effects of digoxin on HIV-1 RNA processing in greater detail, we examined for changes in viral RNA splice site selection (Fig. 4A–C). Using RNA from HeLa rtTA-HIV-Δ*Mls* cells incubated in the presence or absence of digoxin, effects on alternative RNA splicing were analyzed by RT-PCR of the HIV-1 MS (2 kb) mRNA class. Position of the primers is illustrated in Fig. 4A. Upon comparison to control samples (Fig. 4B), we noted that digoxin significantly reduced the level of Rev 2/1 mRNA (generated by the use of SA4c, a, b), while having limited effect on other spliced 2 kb mRNAs. Subsequent densitometry analysis of each MS mRNA species (Fig. 4C) revealed that digoxin induced a 73% loss of Rev 2/1 mRNA levels compared to control samples as well as a slight increase in Tat 1 (generated by the use of SA3). In contrast, other splice modulator drugs such as clotrimazole and flunarizine had no significant effect on HIV-1 MS splice site selection (Fig. S5). These results reveal that digoxin causes selective alterations in the use of viral MS pre-mRNA splice sites, leading to the specific loss of the mRNA species encoding a key HIV-1 regulatory factor, Rev (Fig. 4B and C).

### Digoxin induces loss of Rev expression and reduces cytoplasmic accumulation of US viral RNA

To assess the impact of digoxin's alteration of splice site usage at the protein level, we performed western blots of cell extracts to examine for changes in the viral regulatory factors Rev and Tat. Analysis of Rev (Fig. 4D, top) revealed a profound loss of Rev protein expression levels relative to DMSO control (+) consistent with the reduced level of the corresponding mRNA (Fig. 4B and 4C). This response was achieved without detectable changes in the level of p16 Tat, a Rev-independent isoform encoded by MS RNA, demonstrating selectivity in the responses observed. However, digoxin did reduce expression of p14 Tat (Fig. 4D, bottom), a Rev-dependent isoform produced from SS mRNA. Reduced p14 Tat levels is consistent with both a decrease in Rev expression (Fig. 4D, top) and of SS mRNA (Fig. 3). These observations confirm that digoxin selectively blocks Rev protein production, leading to impaired export of Rev-dependent mRNAs (US and SS) that produce viral structural proteins as well as a subset of regulatory/accessory factors (illustrated in Fig. S1).

**Figure 5:**
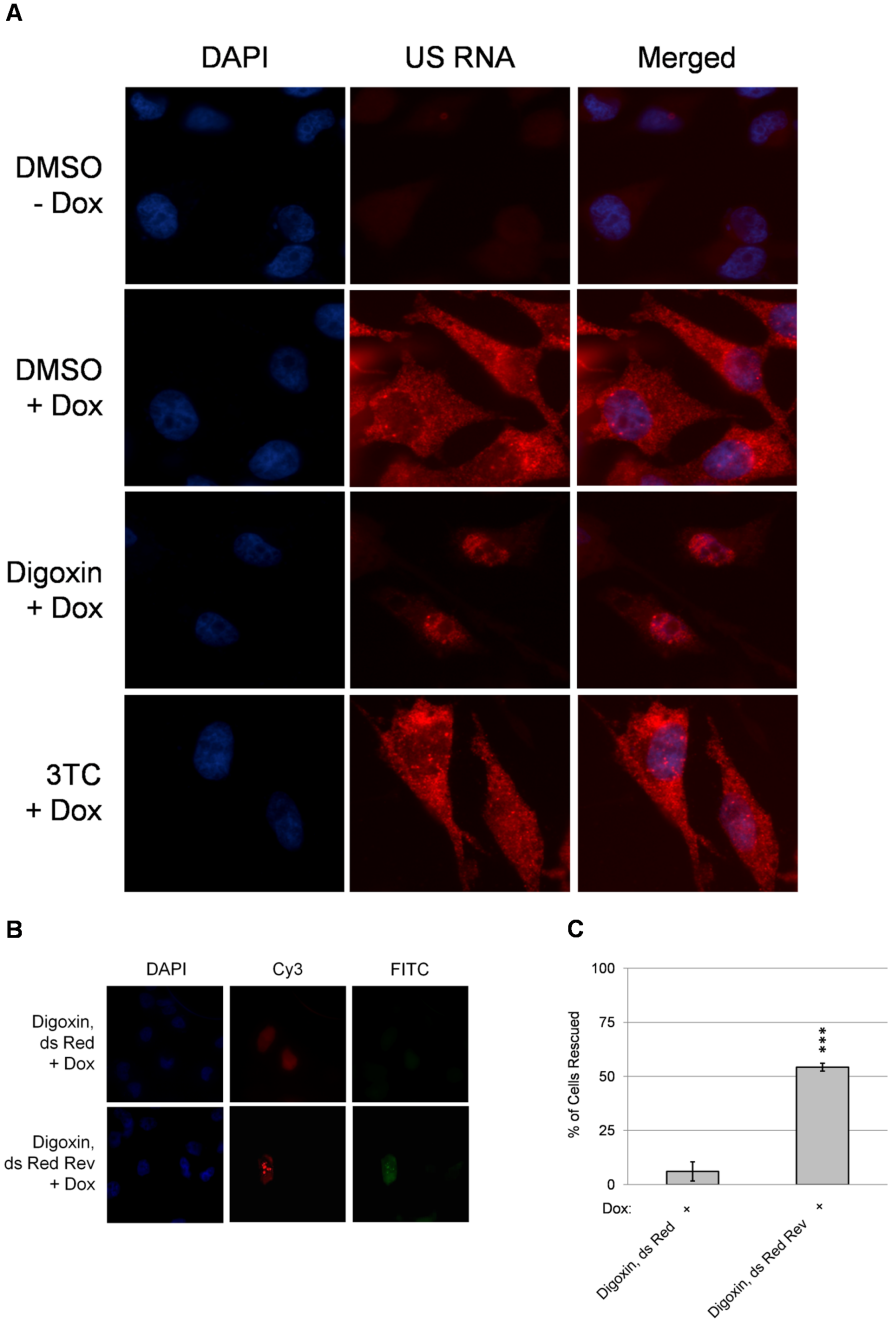
Digoxin blocks cytoplasmic accumulation of HIV-1 US RNA and its effect is partially reversed by expression of Rev *in trans*. (A) To assess the effect of digoxin treatment on viral RNA transport, in situ hybridization of US RNA was performed on HeLa rtTA-HIV-Δ*Mls* cells treated with digoxin, 3TC, or DMSO solvent. After 4 h, viral expression was left uninduced (−) or induced by addition of Dox (+). After 24 h, cells were fixed and incubated with labeled oligonucleotides specific to HIV-1 US RNA (US RNA). After washing to remove unbound probe, cells were stained with DAPI to allow detection of nuclei and images captured at 630× magnification. Shown is a representative sample of the results observed from ≥4 independent experiments. (B) To determine whether Rev expression *in trans* could alleviate the inhibition of Gag protein synthesis by digoxin, cells were transfected with vectors expressing dsRed or a dsRed-Rev fusion protein. At 24 h post-transfection, cells were treated with digoxin for 4 h then Dox was added to induce provirus expression. At 24 h post-doxycycline addition, cells were fixed, DAPI stained, and viewed for co-expression of dsRed (Cy3) and Gag signal (FITC). Shown is a representative sample of the results obtained. Note: intense foci in the nuclei seen in the FITC channel in cells transfected with dsRed-Rev is due to bleed through from the Cy3 channel. To assess the extent of reversing the effects of digoxin by expressing Rev in trans (C), >100 dsRed+ cells were examined in each experiment and the frequency of dsRed+ cells also having Gag+ expression was determined for each condition and displayed as “% of cells rescued”. Error bars of the data are SEM and significant changes from dsRed indicated by asterisk as detailed in “Materials & Methods”.

As further verification that digoxin results in reduced Rev activity, *in situ* hybridization was performed to look for changes in HIV-1 US RNA distribution associated with drug treatment. As shown in Fig. 5A, in the presence of doxycycline alone (DMSO +Dox), signal for HIV-1 US RNA is observed throughout the cell with intense staining at the sites of proviral integration. In contrast, addition of both doxycycline and digoxin results in viral US RNA being predominately restricted to the nucleus (Fig. 5A, Digoxin +Dox,). Treatment of cells with the NRTI, 3TC, had no effect on the distribution of the viral US RNA (Fig. 5A, 3TC +Dox). To determine whether reduction of Rev alone was responsible for the loss of HIV-1 structural protein expression, cells were transfected with control (dsRed) or Rev (dsRed-Rev) expression vectors in the presence of digoxin and Gag protein synthesis monitored (Fig. 5B, C). These assays revealed that *trans* expression of Rev (ds Red Rev) yielded a partial recovery of HIV-1 Gag protein synthesis in comparison to the control vector (ds Red).

### Digoxin inhibits the activity of CLK SR protein kinases and induces modification of a subset of SR proteins

**Figure 6:**
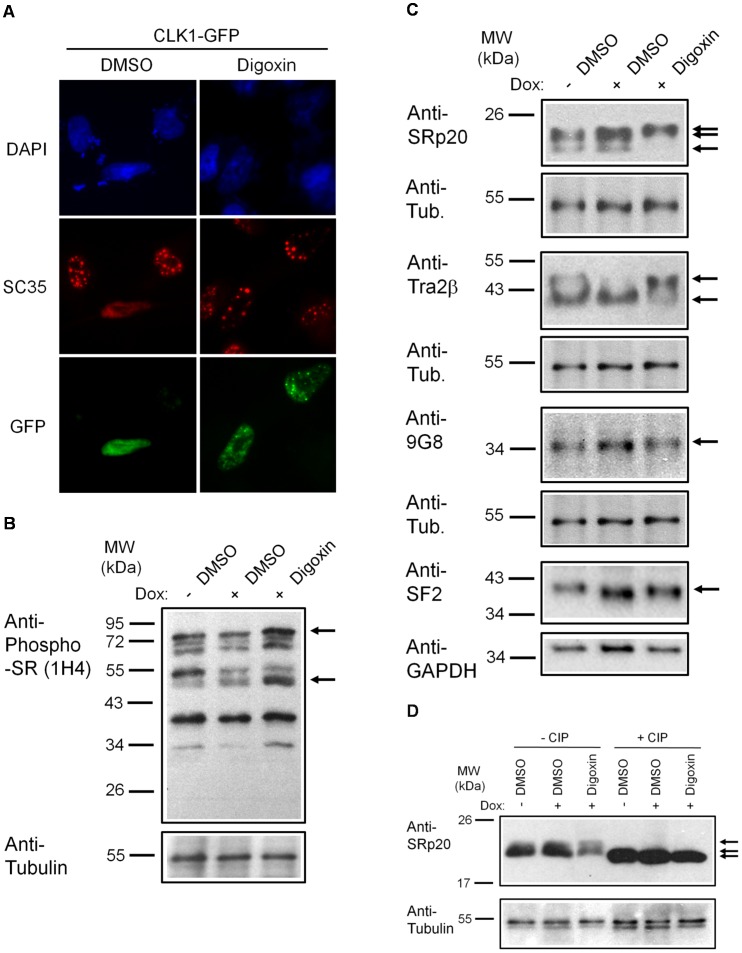
Digoxin alters the activity of SR protein kinases and induces modification of a subset of SR proteins. (A) To assess the effect of digoxin on CLK kinase function, HeLa rtTA-HIV-Δ*Mls* cells were transfected with vectors expressing GFP-tagged CLK1. Twenty-four hours post-transfection, cells were treated with DMSO or 100 nM digoxin. After 24 h of treatment, cells were fixed, stained for SC35/SRSF2 nuclear speckles (Texas Red), and nuclei stained with DAPI. Images are representative of the localization patterns observed from ≥5 independent experiments. Magnification 630×. (B, C, D) Western blot analysis of the effect of digoxin treatment on SR proteins. Lysates were harvested from HeLa rtTA-HIV-Δ*Mls* cells treated with 100 nM digoxin or DMSO and were induced with Dox (+) or left uninduced (−) as described in Fig. 1. Immunoblots were probed by specific antibody for (B) phospho-SR proteins (1H4) or (C) SRp20, Tra2β1, 9G8, or SF2/ASF (SF2). (D) Cell lysates from above were dephosphorylated by treatment with (+) and without (−) calf intestinal alkaline phosphatase (CIP), then immunoblotted with a SRp20-specific antibody. Arrows on the right indicate the protein specie(s) detected and their modified/unmodified isoform(s). Anti-tubulin (Anti-Tub.) blots for each experiment served as an internal loading control for the relative amount of protein lysate per lane. Immunoblots are representative of ≥4 independent trials.

Digoxin inhibits the function of the sodium-potassium (Na^+^/K^+^) ATPase in the plasma membrane resulting in increased intracellular levels of calcium as well as the activation of a number of signaling cascades [38], [39], [44]. How events at the plasma membrane ultimately result in altered HIV-1 RNA processing in this system is not immediately apparent. However, many of the kinase cascades affected by cardiac glycosides have been described to influence alternative RNA splicing [45], [46], [47]. One hypothesis is that digoxin-induced alteration of cellular signaling cascades ultimately affect the activity of factors, such as SR proteins, known to regulate HIV-1 RNA splicing [16], [19]. To test whether any alteration in SR protein function occurred in our experimental system, we first examined the effect of digoxin treatment on SR protein kinases belonging to the CLK family (1–4) [48], [49], [50]. As indicated in Fig. 6A and S6, overexpression of any of these kinases results in a shift in the subnuclear distribution of SR proteins (such as SC35/SRSF2) from being localized to nuclear speckles to being dispersed throughout the nucleus (compare GFP− with GFP+ cells treated with DMSO). Treatment with digoxin reversed the effects of all CLK kinases tested (Fig. 6A and S6, Digoxin); SC35 remained in nuclear speckles in the presence of digoxin despite CLK overexpression, consistent with reduced activity of the transfected kinases.

Impaired activity of a family of SR protein kinases in response to digoxin addition suggests that an alteration in SR protein function underlies the inhibition of HIV-1 replication. To explore this hypothesis, SR proteins were analyzed by western blot of cell lysates (Fig. 6B) for changes in abundance or migration due to drug treatment. Initial analysis of phospho-SR proteins by 1H4 antibody determined that digoxin treatment increased the levels of at least two phospho-SR proteins (Fig. 6B): increasing SRp55 and moderately increasing SRp75 relative to DMSO controls (+/−). No consistent changes in the overall phospho-SR protein levels were observed in the presence or absence of HIV-1 expression by this antibody. To further explore specific members of SR proteins affected by digoxin, we performed western blot analysis on a panel of SR proteins with specific antibodies to SRp20, Tra2β, 9G8, and SF2/ASF (Fig. 6C). Recent work [51] demonstrated that treatment with digitoxin (another cardiac glycoside) induced marked alterations in SRp20 and Tra2β abundance. Consistent with the selective effect of a cardiac glycoside on a subset of SR proteins, we observed that SRp20 (Fig. 6C) underwent a shift to a higher MW species upon digoxin treatment compared to DMSO-treated cells (+/−). Treatment of extracts with alkaline phosphatase confirmed that the shift observed in SRp20 was due to hyperphosphorylation of the protein (Fig. 6D). In the case of Tra2β (Fig. 6C), digoxin treatment increased the level of a high MW form of Tra2β that was reduced upon induction of HIV-1 (+ Dox) compared to control (−Dox). However, alkaline phosphatase had no effect on the higher MW forms of Tra2β blots induced by digoxin treatment (data not shown). Analysis of other SR proteins, 9G8 and SF2/ASF (Fig. 6C), showed little or no change in levels or MW upon digoxin treatment. These data are consistent with the recent work of Anderson et al. [51] in that only a subset of SR proteins are affected by digoxin treatment, suggesting that at least one or a combination of these splice factors play a critical role in mediating the change in HIV-1 RNA processing or expression.

### SRp20 overexpression mimics the effects of digoxin on HIV-1 RNA processing

**Figure 7:**
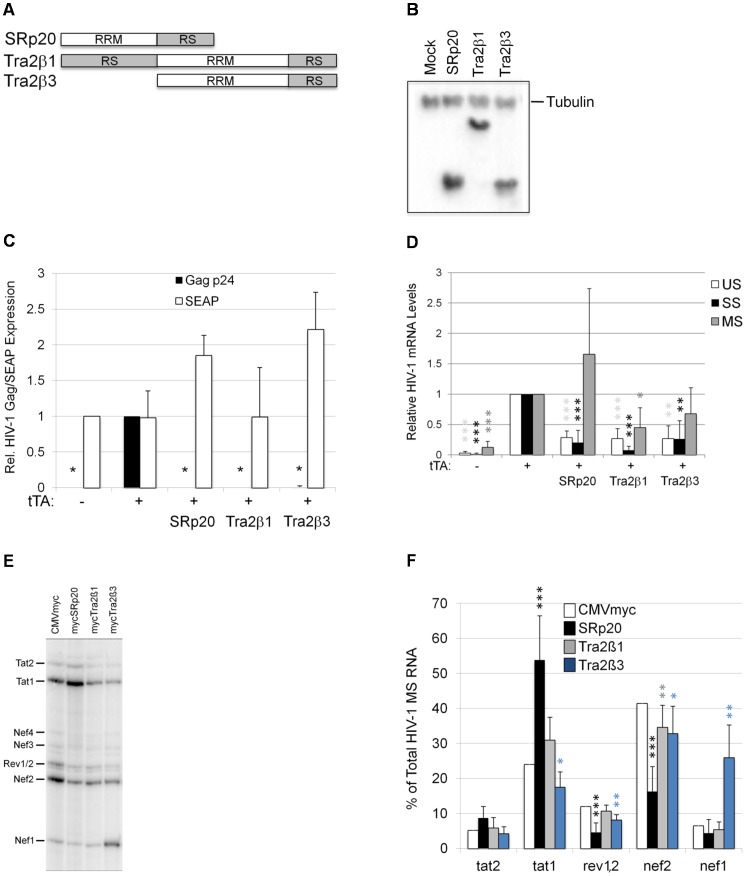
Overexpression of SRp20, Tra2β1, or Tra2β3 suppress HIV-1 expression. (A) Diagram of SRp20, Tra2β1, and Tra2β3 protein domain structure. (B) Cells were transfected with the vectors CMVmyc (mock), CMVmyc SRp20 (SRp20), CMVmyc Tra2β1 (Tra2β1), or CMVmyc Tra2β3 (Tra2β3). Forty-eight hours post-transfection, cells were harvested, lysed in RIPA buffer, and proteins analyzed by western blot with anti-myc antibody followed by anti-tubulin (Tubulin) antibody to indicate loading. (C) HeLa rtTA-HIV-Δ*Mls* cells were transfected with (+) (or without, −) CMVtTApA (tTA) to activate the endogenous HIV-1 provirus along with CMV PLAP and either CMVmyc, CMVmyc SRp20, CMVmyc Tra2β1, or CMVmyc Tra2β3. Forty-eight hours post transfection, cell media was harvested and assayed by p24^CA^ ELISA for expression of HIV-1 Gag (black) and production of SEAP (white). Results are averaged from 6 independent assays, error bars are SEM, and asterisks indicate values deemed significant from CMVmyc (+tTA) as detailed in “Materials & Methods”. (D) In parallel, total RNA was extracted from transfected cells and the abundance of individual viral RNA (unspliced, US; singly spliced, SS; multiply spliced, MS) were determined by qRT-PCR. Shown are results averaged from 6 independent assays, error bars are SEM, and asterisks indicate significance as noted above. (E, F) MS HIV-1 RNAs from transfected cells were amplified by RT-PCR as described in Fig. 4. Amplicons were fractionated on urea-PAGE gels and a representative gel is shown (E). Relative abundance of individual MS viral RNA species were quantitated and graphed (F). Data was averaged from >5 independent assays, errors bars are SEM, and asterisks indicate statistical significance as described above.

The increased SRp20 phosphorylation or changes in Tra2β modification in response to digoxin raised the possibility that the alterations in HIV-1 RNA splicing could be attributed to increased activity of either factor. To test this hypothesis directly, HeLa rtTA-HIV-Δ*Mls* cells were transfected with vectors expressing these factors and their effects on viral structural protein and RNA accumulation were assessed (Fig. 7). To ensure that only cells taking up DNA expressed the HIV-1 provirus, cells were also co-transfected with plasmids expressing the TetO activator, tTA, to induce provirus expression, and secreted enzyme alkaline phosphatase (SEAP) as an indicator of global effects on gene expression. As shown in Fig. 7C, detection of HIV-1 Gag by p24^CA^ ELISA was dependent upon transfection with tTA (see −tTA vs. +tTA). Transfection of SRp20 or either isoform of Tra2β (Tra2β1 and Tra2β3) resulted in a marked reduction in Gag protein expression with unchanged or increased expression of SEAP. Subsequent analysis of viral RNA accumulation indicated that each factor functioned in a different manner. qRT-PCR of each of the HIV-1 mRNAs (Fig. 7D) determined that SRp20 overexpression resulted in reduced accumulation of both US and SS viral RNAs with a trend towards increased MS RNA levels. In contrast, overexpression of either isoform of Tra2β resulted in reduced accumulation of all HIV-1 mRNAs. Subsequent analysis of splice site selection within the MS class of viral RNAs revealed distinct differences in how these factors affected HIV-1 MS RNA splicing (Fig. 7E, F). Similar to digoxin, SRp20 overexpression induced a shift in splice site usage that resulted in increased Tat1 accumulation while reducing Rev1/2 and Nef2 levels. In contrast, Tra2β1 overexpression elicited little change in splice site selection while Tra2β3 overexpression induced a marked accumulation of Nef1, generated by splicing the first 5'ss of HIV-1 to the last 3'ss of the virus. Taken together, the response to SRp20 overexpression is most similar to that observed upon digoxin treatment.

## Discussion

**Figure 8:**
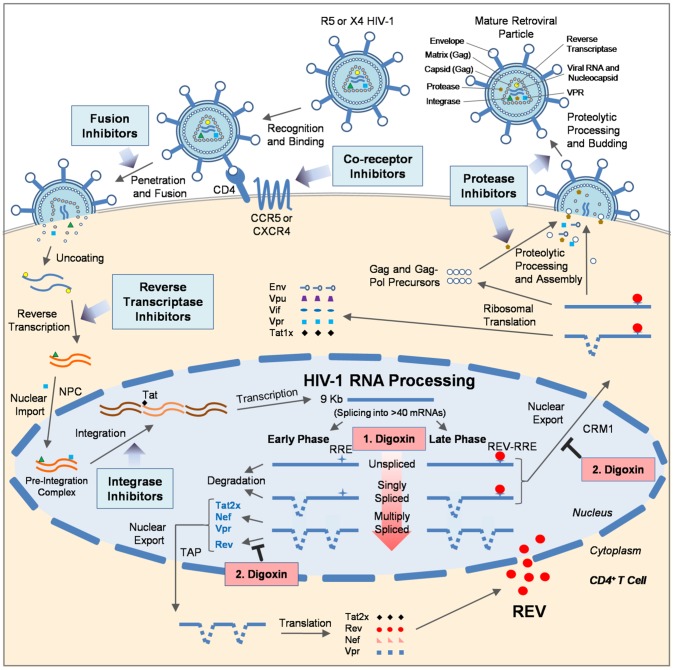
Digoxin inhibits HIV-1 replication at a new stage of the virus lifecycle. The diagram outlines the current targets of HIV-1 ART/HAARTs which include inhibitors of viral entry (co-receptor and fusion) and all viral enzymes: reverse transcriptase, integrase, and protease. This study demonstrates that digoxin is a potent inhibitor of HIV-1 replication that perturbs RNA processing, a new and viable target of the virus lifecycle. Digoxin inhibits HIV-1 replication by altering specific pre-mRNA splicing events under the control of the host. This includes (1) inducing oversplicing of HIV-1 pre-mRNA (depicted by an increasingly red arrow), which reduces both unspliced (US) and singly spliced (SS) mRNA levels, and (2) altering the use of pre-mRNA splice sites within multiply spliced (MS) mRNA, leading to the reduction of mRNA (and protein) encoding a key HIV-1 regulatory factor, Rev. Loss of Rev further impacts the expression of viral proteins from incompletely-spliced mRNA (US and SS) by impeding nuclear export of these RNAs. The reduction of incompletely-spliced viral mRNAs by both of these mechanisms disrupts the synthesis of a subset of HIV-1 regulatory and accessory factors as well as structural proteins necessary for new virion assembly. This study demonstrates that digoxin, a member of the cardiac glycoside family of drugs, represents a novel class of HIV-1 inhibitors targeting viral RNA processing.

Despite the success of ART/HAART, there are many caveats with current HIV-1 therapies, including the emergence of drug resistant forms of HIV-1, high cost, and toxicity [1], [2], [10]. New drugs with improvement in these profiles and novel mechanisms of action are necessary [1], [2], [9]. A number of strategies have targeted HIV regulatory and accessory proteins to date, but most remain under development [9], [52]. It is unclear whether disrupting cellular processes essential for HIV-1 replication can yield alternative therapies without significant cellular toxicity. However, a number of existing therapies for other human diseases (e.g. heart disease, cancer, and dementia) do work by altering host protein function and are well tolerated [53], [54], [55], [56]. In this report, we demonstrate a novel and alternative use of the FDA-approved cardiovascular drug, digoxin, as an anti-HIV-1 therapeutic (summarized in Fig. 8). More importantly, digoxin was found to inhibit virus replication by a novel mechanism, inducing oversplicing of HIV-1 RNA (Figs. 3, S3, 4, and S4D)—a stage of the virus lifecycle not targeted by current HIV-1 inhibitors and under host cell control. Digoxin achieves this effect by altering the splicing of HIV-1 RNA, reducing accumulation of two classes of viral mRNA (US and SS; Figs. 3, S3, S4D) that encode structural proteins essential for new virion assembly (Gag, Gagpol, and Env; Fig. 1). In addition, digoxin selectively inhibits expression of the HIV-1 regulatory factor Rev through specific alteration of viral RNA splice site use without affecting the expression of other viral proteins (p16 Tat; Fig. 4). While digoxin induced a 73% reduction in Rev2/1 RNA accumulation, it also increased MS viral RNA levels ∼3 fold (Fig. 3). Combined, these alterations may not account for the complete loss of Rev protein observed, suggesting the possibility that digoxin may have effects beyond the changes in viral RNA processing detected. The loss of Rev further impairs expression of incompletely-spliced viral mRNAs (US and SS) by preventing Rev-mediated export of RNAs to the cytoplasm (Fig. 5A) for translation into respective viral structural proteins (Gag, Gagpol, and Env) and regulatory/accessory factors (p14 Tat, Vif, Vpr, and Vpu) (Figs. 1, 2, S2, 4D). Furthermore, the effects were achieved at concentrations of digoxin that did not impact HeLa, SupT1, and PBMC cell viability relative to control treatments (Figs. 1, 2, and S4). Rev expression *in trans* (Fig. 5B–C) only partially reversed the effects of digoxin, indicating that loss of Rev alone is not sufficient to explain the full effect of digoxin. Rather, in light of the demonstration that Rev acts primarily on newly synthesized viral RNA [57], the enhanced processing of the viral RNA induced by digoxin may result in the incompletely-spliced HIV-1 RNAs having too short a half-life to be engaged by Rev even when Rev is present. In summary (Fig. 8), digoxin selectively impairs HIV-1 replication at two levels: (1) through global alterations in the efficiency of HIV-1 RNA processing and (2) blocking export of incompletely-spliced viral RNAs to the cytoplasm.

Digoxin and other cardiac glycosides are known to bind the Na^+^/K^+^-ATPase pump in the plasma membrane, initiating the activation of multiple signaling cascades that result in increased intracellular calcium concentrations as well as signaling of Src, AKT, and MAPK kinases [44], [58], [59]. How this response initiated at the cell membrane can alter RNA splicing was not immediately clear. In light of the observed changes in HIV-1 RNA processing, we initially focused on factors known to modulate these events: SR proteins [60], [61], [62], [63]. Consistent with the findings of Anderson et al. [51], our results reveal that a subset of SR proteins (SRp20, Tra2β, SRp55, and SRp75) are altered as well as the function of a number of SR protein kinases (CLKs 1–4) upon digoxin addition (Fig. 6). In the work of Anderson et al. [51], only a subset of the exons examined were affected by treatment with digitoxin, suggesting that the response is not a general perturbation of host RNA splicing but is more selective. Since the modifications of SRp20 or Tra2β1 detected might increase their activity, we subsequently examined the impact of overexpression of both factors on HIV-1 RNA processing (Fig. 7). While the three factors tested (SRp20, Tra2β1, and Tra2β3) all elicited a marked reduction in HIV-1 Gag synthesis upon overexpression, analysis of the effects on viral RNA splicing determined that the basis for the response was markedly different. Of the three factors tested, overexpression of SRp20 most closely mimics the changes induced by digoxin; reducing accumulation of US and SS viral RNAs while trending towards increased MS RNA abundance. Furthermore, SRp20 induced increased accumulation of Tat1 and reduced Rev1/2 mRNA levels as observed with digoxin. The response documented here differs significantly from those induced by overexpression of SC35, SRp40, 9G8, and SF2/ASF previously reported [18], [20], [31]. In these studies, overexpression of SC35, SRp40, or 9G8 resulted in almost exclusive formation of MS RNA encoding Tat (Tat1), while SF2/ASF increased usage of the splice sites for Vpr. However, the effects of these factors on HIV-1 RNA accumulation and expression differ among published reports: one indicates that SF2/ASF, SC35, or SRp40 overexpression increases US viral RNA accumulation [18] while another showed marked reduction of all viral RNAs with only SF2/ASF significantly decreasing Gag expression [31]. None of these reports demonstrated selective alterations in Rev1/2 RNA abundance comparable to digoxin or SRp20 overexpression reported here. Future efforts will be focused on understanding how SRp20 achieves this response on HIV-1, through either direct interaction with sites on the viral RNA and/or manipulation of abundance/activity of other host factors.

In contrast to the effects of SRp20, overexpression of Tra2β1 and Tra2β3 reduced levels of all viral RNAs (Fig. 7D) while only Tra2β3 altered splice usage to favor Nef1 (Fig. 7E, F). The difference in activity of Tra2β1 and Tra2β3 is of particular interest since both share a common RRM domain as well as a C-terminal RS domain (Fig. 7A) and interact with a common set of SR proteins [64]. However, previously analyses had indicated that Tra2β3 had limited or no ability to modulate splicing of a number of RNA substrates tested [65]. Our demonstration that the two Tra2β isoforms have quite distinct effects on splice usage in the context of HIV-1 RNA splicing suggests that variation in abundance of these two isoforms of Tra2β is likely to yield quite distinct effects on host cell RNA splicing. The response seen upon Tra2β3 overexpression is most similar to alterations induced upon mutation of the exon splicing enhancer (GAR) adjacent to SA5. Previous studies had determined that reduced function of GAR resulted in increased accumulation of spliced RNA corresponding to Nef1 [66], [67], raising the possibility that Tra2β3 functions by interfering with GAR function.

Our determination that digoxin can alter the equilibrium in viral RNA processing demonstrates that this step of the virus lifecycle can be manipulated to block HIV-1 replication. In principle, targeting host factors essential for HIV-1 replication offers the promise of broad spectrum activity against multiple viral strains and a reduced potential of resistance. Although digoxin has potent effects on HIV-1 in our assays, its use in the treatment of cardiovascular conditions has a narrow therapeutic dose range of 0.5–2.0 ng/mL (max. 5 nM) with higher doses yielding increased toxicity (including death) [38], [39]. Our experiments using the stably transduced HeLa rtTA-HIV-Δ*Mls* and 24ST1NLESG cell lines determined that complete suppression of HIV-1 gene expression requires concentrations of digoxin (IC_90_ = 100 nM, Fig. 1C; IC_90_ = 370 nM, Fig. S4C, respectively) well above what is compatible for use in humans. However, our subsequent studies using PBMCs showed that reduced doses of digoxin are sufficient to achieve a significant response (IC_90_ = 25 nM, Fig. 2A, B). In experiments using HIV infected patient PBMCs, doses as low as 2 nM strongly suppressed HIV-1 replication (Fig. 2C, D). The differences in the dose of digoxin required to achieve a measurable response between the various assays might reflect differences in the ability to activate the signaling cascade initiated by the binding of digoxin to the Na^+^/K^+^ ATPase at the cell surface [44]. Given the transformed nature of both HeLa and SupT1 cells, it is not unexpected that portions of this cascade may be altered relative to PBMCs. Alternatively, differences in the response of the different cell types (HeLa/SupT1 vs. PBMCs) to digoxin may reflect the nature of the assay itself. In the experiments using the stably transduced cell lines (HeLa/SupT1), >90% of the cells are expressing viral proteins upon induction and, hence, inhibition would require significant alterations in HIV-1 RNA processing/protein synthesis. In contrast, for PBMCs, detection of Gag expression is dependent upon the exponential amplification of the virus in the culture. In this context, even small perturbations in HIV-1 replication will result in significant differences over multiple rounds of replication. The benefit is that doses of digoxin within the therapeutic range were able to suppress HIV infection. Better responses might be achieved using derivatives of digoxin with improved activity and a better therapeutic index [44], [58]. The determination that digoxin, acting through the Na^+^/K^+^-ATPase (a plasma membrane receptor), can suppress HIV-1 gene expression suggests that its downstream effectors might also prove to be therapeutic targets. In addition, compounds which mimic digoxin's effect on CLKs and/or SR protein function could prove equally capable of altering HIV-1 RNA processing. Several compounds affecting CLK function (TG003 and chlorohexidine) have already been described [36], [68], and we recently demonstrated that chlorhexidine (but not TG003) inhibits HIV-1 gene expression [33]. Recent studies [59], [69] have identified multiple kinases mediating the effect of cardiac glycosides in transformed cells. Determining which of these kinases is responsible for mediating digoxin's effect on HIV-1 RNA processing would be useful in developing a more targeted approach to manipulating viral gene expression. However, the demonstration that digoxin can inhibit HIV-1 replication through a novel mechanism without significant toxicity to the host cell serves as proof that this strategy is viable and could be used in junction with existing treatments for better control of this infection.

## Materials and Methods

### Screening of splice modulator drugs

Screening of drugs for effects on HIV-1 RNA processing was performed using the HeLa rtTA-HIV-Δ*Mls* cell line containing an inducible Tet-On HIV-1 provirus [40], [41] as described in our previous study [33]. Activation of virus gene expression in these cells was achieved by addition of doxycycline (Dox) or transfection of plasmid expressing tTA. In drug screens, cells were seeded one day prior in IMDM containing 10% FBS, 1X Pen-Strep, and 1X Amphotericin B (Wisent Corporation) while drugs were solubilized to ∼1000X of its final treatment concentration in DMSO. Next, cells were treated for 4 h with 100–200 µL of drugs pre-diluted to ∼25X of its final concentration in Opti-MEM (Invitrogen, #31985070) and HIV gene expression was induced with Dox (2 µg/mL). After ∼24 h of drug treatment, cells and media were harvested. To monitor effects of drug treatments, p24^CA^ ELISA, western blotting, and RNA analyses were performed as described below. Cell viability was assessed using biochemical (XTT assay; Sigma-Aldrich, #TOX2) and/or physical (trypan blue exclusion; Invitrogen, #15250-061) assays.

### Ethics statement

Written informed consent was obtained from volunteer blood donors in accordance with the guidelines for conduct of biomedical research at the University of Toronto, and all experimental protocols were approved by the University of Toronto institutional review board.

### HIV-1 growth in PBMCs

Human primary cells were obtained for experiments either from healthy volunteer blood donors (uninfected with HIV) or drug-naïve HIV-infected individuals. For infection experiments, PBMCs were isolated, infected with HIV-1 (BaL), and cultured as described previously [33]. Cells were treated with drugs pre-diluted in RPMI in the same manner as described above. Every 3–4 days, 0.5 mL of media was harvested for p24^CA^ ELISA and replaced with 0.5 mL of fresh R-10 medium containing fresh drug treating 1 mL (∼1.5X final with fresh and decayed drug). The effect of drugs on cell health was assessed in parallel by XTT and/or trypan blue assays.

For experiments using HIV-infected patient samples, PBMCs were first depleted of CD8^+^ T cells using Dynabeads CD8 (Invitrogen, #111.47D) as outline by manufacturer. Remaining cells were then activated by treatment with anti-CD3 anti-CD28 antibodies (Bio Legend #302914 and 317304, respectively; 1 µg/ml of each) as well as 50 U/ml of IL-2 (BD Pharmingen #554603) in the presence or absence of indicated drugs as mentioned above. Media (0.5 ml) was collected every 3–4 days and replaced with fresh media (0.5 ml) containing 20 U/ml of IL-2 and fresh drug. Effect of compounds on cell viability was monitored in parallel by XTT assay and expressed relative to control (DMSO) treated cells. HIV-1 growth in cultures was monitored by p24^CA^ ELISA of cell supernatants.

### Quantitation of HIV-1 mRNA levels and localization

RNA was extracted from cells by Aurum Total RNA Mini Kits (Bio-Rad, Cat. #732-6820). Purified RNA was reverse transcribed using M-MLV (Invitrogen, Cat. No. 28025-013) and resulting cDNAs were used to quantitate HIV-1 mRNA levels by qRT-PCR as described [33]. To monitor for changes in HIV-1 US RNA subcellular distribution in response to digoxin, cells were treated with digoxin, 3TC, or DMSO solvent for 4 h and then viral gene expression was induced by addition of Dox. After 20 h, cells were fixed in 3.7% formaldehyde-1XPBS. Cells were permeabilized by treatment with 70% EtOH, then rehydrated in hybridization buffer (10% formamide, 2XSSPE). Hybridization was performed using a mixture of 48 Quasar 570-labelled oligonucleotides spanning the MA, CA, and nucleocapsid (NC) regions of HIV-1 as detailed by the supplier (Biosearch Technologies). Following washing to remove unbound probe, nuclei were stained with DAPI and images acquired using a Leica DMR microscope at 630× magnification.

### Analysis of HIV-1 splice site usage

The effect of drugs on HIV-1 splice site usage within the 2 kb, MS RNA class was analyzed by performing RT-PCR of cDNA obtained from RNA purified and reverse transcribed as previously described [33].

### Effect of digoxin on CLK function

HeLa rtTA-HIV-Δ*Mls* cells were transfected with vectors expressing GFP-tagged CLK1, CLK2, CLK3, or CLK4. Twenty-four hours post-transfection, cells were treated with either digoxin or DMSO for 24 h, fixed, processed, and analyzed by immunofluorescence microscopy [33]. Effects on SC35 localization was assessed using a mouse anti-SC35 antibody (BD Pharmingen, #556363) and a secondary Texas Red-conjugated donkey anti-mouse IgG antibody (Jackson Immunoresearch, #715-075-151), while nuclei were stained with DAPI.

### Analysis of HIV-1 and SR protein expression

To monitor HIV-1 gene expression or virus replication (Gag synthesis), cell culture supernatants were assayed by a HIV-1 p24^CA^ antigen capture assay kit (AIDS & Cancer Virus Program, NCI-Frederick, Frederick, MD USA). Media harvested from PBMC cultures infected with HIV-1 (BaL) were diluted ∼250-fold (or as needed) prior to performing this assay. For analysis of HIV-1 and SR protein expression by Western blot, cells were solubilized in RIPA buffer, quantitated by Bradford assay, and run on 8, 10, or 12% SDS-PAGE under reducing conditions, and then transferred to PVDF. Normally, 25–30 µg of protein was loaded, blots blocked in either 5% Milk-T (5% skim milk, 0.05% tween-20, 1XPBS) or 3% BSA-T for 1 h at room temperature (RT) according to the antibody diluent used, and blots incubated with antibody at RT for ∼2.5 h, unless otherwise specified. Specific antibodies and conditions used for Tat, anti-tubulin, and isotype-specific HRP-conjugated antibodies were used as described [33]. Additional antibodies and conditions used in this study include a mouse anti-p24 supernatant from hybridoma 183 (provided by M. Tremblay, Laval University): 1/10^th^ dilution in PBS-T incubated for 1 h at RT, blocked in 5% Milk-T overnight at 4°C. Mouse anti-gp120 purified supernatant from hybridoma 902 (obtained through the AIDS Research and Reference Reagent Program, Division of AIDS, NIAID, NIH: Hybridoma 902 (anti-gp120) from Dr. Bruce Chesebro): 1/10^th^ dilution in PBS-T incubated normally or O/N at 4°C, blocked in 3% BSA-T at RT for 2.5 h. Mouse anti-Rev (Abcam, #ab85529): 1/1000^th^ dilution in 3% BSA-T incubated O/N at 4°C, loaded with 30–40 µg of protein. Mouse anti-phospho-SR (1H4) (Invitrogen, #33-9400): 1/5000^th^ dilution in 3% BSA-T, blocked for ∼2.5 h at RT or overnight at 4°C. Rabbit anti-Tra2β (Abcam, #ab3135353): 1/10,000^th^ dilution in 3% BSA-T incubated for 1.5 h at RT. Rabbit anti-9G8 serum (Znk1.4): 1/3000^th^ dilution in 5% Milk-T. Mouse anti-SRp20 (Invitrogen, #334200), 1/1000^th^ dilution in 3% BSA-T, loaded with 20 µg of protein. Generally, Western Lightning-ECL (Perkin-Elmer, #NEL101) but for anti-Rev, -Tat, and -gp120 blots, Western Lightning Plus-ECL (#NEL105) were used for development of signals onto autoradiography film. In addition, phosphatase inhibitors (e.g. 10 mM sodium fluoride, 2 mM sodium orthovanadate) were added to solutions for SR protein analyses. Lastly, SR protein phosphorylation was confirmed through treatment of ∼20 µg of cell lysate with 20 U of calf intestinal alkaline phosphatase (NEB, #M0290S) for ∼45 minutes at 37°C prior to western blot analysis.

### Effect of SR protein and Rev overexpression on HIV-1 gene expression

To assess effects of protein overexpression, cells were transfected in the presence or absence of the tTA expression vector, CMV PLAP (expressing SEAP/alkaline phosphatase), and either empty vector (CMVmyc pA), CMVmyc SRp20, CMVmyc Tra2β1, or CMVmyc Tra2β3 using polyethylene imine (PEI). At 48–72 h post-transfection, cells and media were harvested. To monitor effects of these manipulations, p24^CA^ ELISA, western blotting, and RNA analyses were performed as described previously [33].

To assess the ability of expression of Rev *in trans* to rescue the synthesis of HIV-1 Gag in the presence of digoxin, cells were transfected as described above with plasmids expressing either dsRed or a dsRed-Rev fusion. At 24 h post-transfection, cells were treated with digoxin for 4 h then HIV-1 expression was induced for 20 h by addition of doxycycline. Cells were subsequently fixed and examined by immunofluorescence for co-expression of Gag and dsRed signal using a Leica DMR microscope.

### Statistical analysis

Data was analyzed using Microsoft Excel and expressed as means ± standard error of the mean (SEM). Differences between two groups of data (i.e. drug treatment vs. DMSO (+Dox) control, drug treatment vs. DMSO (+HIV), or transfected factor vs. mock vector (+tTA) were compared by Student's *t*-test (two-tailed). Statistical significance of results are indicated on each graph as follows: p value<0.05, *, p value<0.01, **, and p value<0.001, ***, unless otherwise indicated.

## Supporting Information

Figure S1 **Pattern of HIV-1 RNA splicing.** Shown at the top is the organization of the HIV-1 proviral genome indicating the position of the multiple 5′ splice donor sites (SD1–4) and 3′ splice acceptor sites (SA1–3; SA4c, a, and b; SA5; and SA7) used in splicing of pre-mRNA. In the middle is an illustration of the alternatively spliced RNAs generated by processing of the HIV-1 genomic RNA. Indicated are the common (open boxes) and alternative exons (closed boxes) used in the generation of the SS (4 kb) and MS (1.8 kb) viral RNAs. At the bottom is a list of the nomenclature used to refer to the exon composition of the individual RNAs generated for both the SS and MS classes of HIV-1 RNAs.
(TIF)

Figure S2 **Digoxin inhibits HIV-1 (BaL) gene expression in PBMCs at day 3.** Isolated PBMCs were infected with R5 HIV-1 BaL at a MOI of 10^−2^ in the presence of indicated doses of digoxin as described in Fig. 2. Equal concentrations of DMSO were present in each treatment. After 3 days, media was harvested for analysis of HIV-1 Gag protein expression by p24^CA^ ELISA. Plotted data was averaged from ≥3 experiments, error bars are SEM, and asterisks indicate significant differences in digoxin treatment from DMSO control as detailed in “Materials & Methods”.
(TIF)

Figure S3 **Digoxin alters HIV-1 RNA processing in a dose-dependent manner.** The effect of increasing concentrations of digoxin on HIV-1 mRNA levels was assessed by qRT-PCR of cells treated with drug or DMSO as described in Fig. 1. Equal concentrations of DMSO were present in each treatment. Primers used in the amplification are described in Fig. 3 and “Materials & Methods”. The abundance of HIV-1 unspliced (US), singly spliced (SS), and multiply spliced (MS) mRNAs are shown relative to DMSO (+) control as indicated on the left. The housekeeping gene, β-actin, served as an internal loading control for the normalization of data. Data was averaged from ≥4 experiments, error bars are SEM, and asterisks indicate significant changes in treatments from DMSO (+) control as described in “Materials & Methods”.
(TIF)

Figure S4 **Effect of digoxin on HIV-1 expression and RNA processing in a chronic-infected human T cell line.** (A) Schematic diagram of the HIV-1 provirus vector (NLE^-^S-G) used to generate the stably transduced SupT1 human T cell line, 24ST1NLESG [43]. (B) Induction of HIV-1 gene expression in 24ST1NLESG cells by PMA. Cells were incubated in the presence (+) or absence (−) of PMA (1.8 µM) and Gag expression was assayed 24 h after PMA addition by p24^CA^ ELISA. Data was averaged from ≥11 experiments, error bars are SEM, and asterisks indicate significant change from DMSO (-PMA) as detailed in “Materials & Methods”. (C) Analysis of the effect of digoxin on HIV-1 gene expression in 24ST1NLESG cells. Cells (seeded at 1×10^6^ cells per mL in RPMI complete medium) were treated with indicated concentrations of digoxin for 4 h (prepared as described in “Materials and Methods” but in RPMI complete medium) and then HIV-1 gene expression was induced by addition of PMA. Equal concentrations of DMSO solvent were present in each treatment. After 24 h, cell media was harvested for quantitation of Gag expression by p24^CA^ ELISA and cell viability was assessed in parallel by XTT assay (grey circles, secondary y-axis) as indicated. Shown is data averaged from ≥3–4 experiments, error bars are SEM, and asterisks indicate significant changes of treatment from DMSO (+) control as described in “Materials & Methods”. (D) Assessment of the effect of digoxin on HIV-1 mRNA accumulation. Cells were treated as outlined above, total RNA was extracted, and the abundance of US, SS, and MS forms of HIV-1 mRNA were quantitated by qRT-PCR as described in Fig. 3. Data was averaged from ≥5 experiments, error bars are SEM, and asterisks indicate significant changes in treatments from DMSO (+) controls as described in “Materials & Methods”.
(TIF)

Figure S5 **Clotrimazole and flunarizine do not affect HIV-1 pre-mRNA splice site usage.** Effects of clotrimazole and flunarizine on HIV-1 splice site use were assayed by RT-PCR of the 2 kb, MS class of HIV-1 mRNA. Primers used to amplify HIV-1 MS mRNA species are described in Fig. 4 and “Materials & Methods”. This graph summarizes the effects of clotrimazole (white), flunarizine (gray), and control treatments (black) on the levels of each MS mRNA species (x-axis) relative to the total HIV-1 MS mRNA (y-axis), displayed as a fraction. Data was averaged from multiple experiments and error bars are SEM, and statistical significance calculated as described in “Materials & Methods”.
(TIF)

Figure S6 **Digoxin alters the activity of CLK SR protein kinases.** To assess the effect of digoxin on CLK kinase function, HeLa rtTA-HIV-Δ*Mls* cells were transfected with vectors expressing GFP-tagged CLK1, CLK2, CLK3, or CLK4 as described in Fig. 6. Twenty-four hours post-transfection, cells were treated with (A) DMSO (control) or (B) 100 nM digoxin. After 24 h of treatment, cells were fixed, stained for SC35/SRSF2 nuclear speckles (Texas Red), and nuclei stained with DAPI. Images are representative of the localization patterns observed from ≥5 independent experiments. Magnification 630×.
(TIF)
