# Supplementary figures and images for: Digoxin Suppresses HIV-1 Replication by Altering Viral RNA Processing
Source: PLoS Pathog. 2013 Mar 28;9(3):e1003241. doi: 10.1371/journal.ppat.1003241 (PMC3610647; doi:10.1371/journal.ppat.1003241)

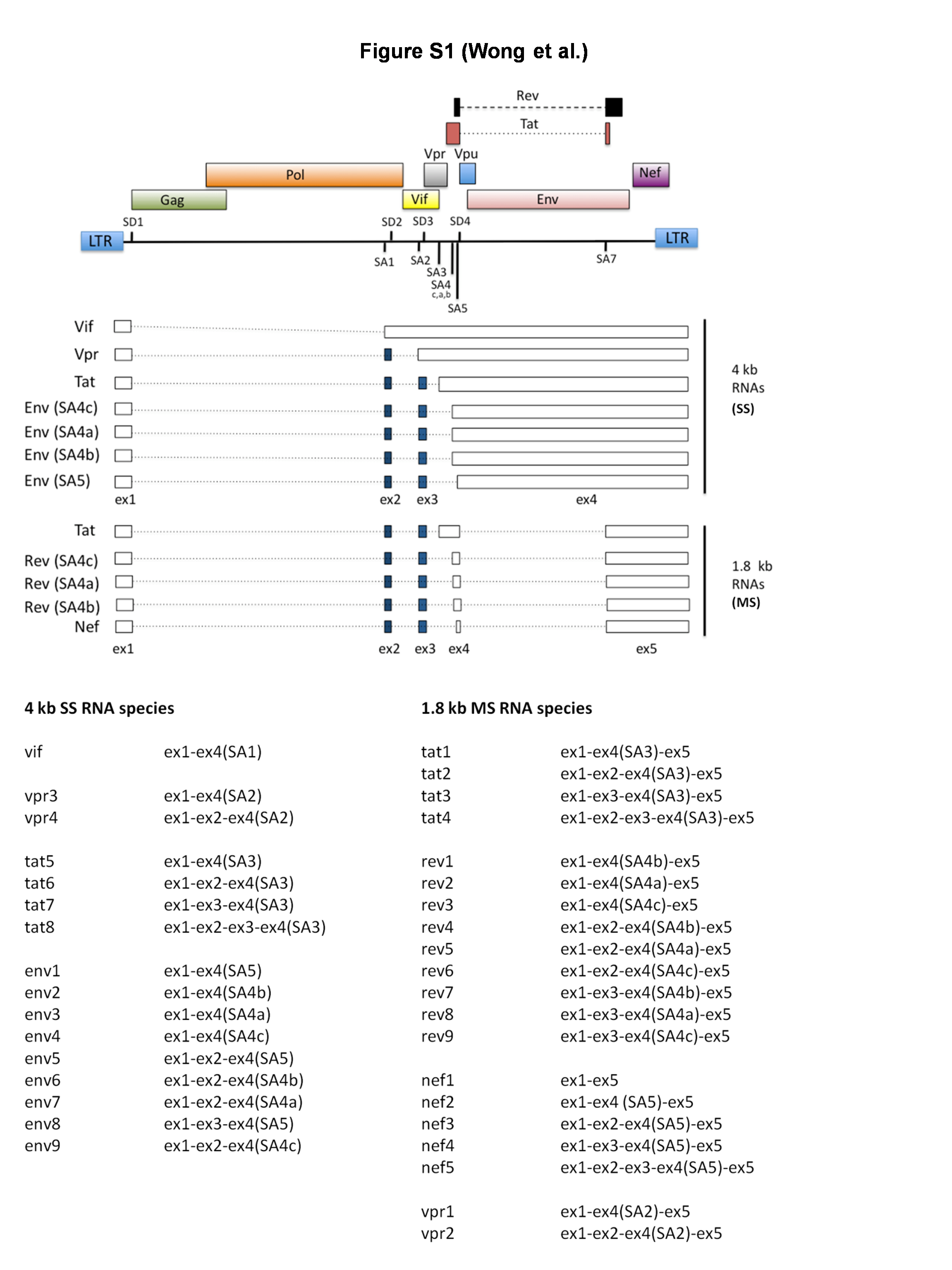

Supplement: Figure S1 — Pattern of HIV-1 RNA splicing. Shown at the top is the organization of the HIV-1 proviral genome indicating the position of the multiple 5′ splice donor sites (SD1–4) and 3′ splice acceptor sites (SA1–3; SA4c, a, and b; SA5; and SA7) used in splicing of pre-mRNA. In the middle is an illustration of the alternatively spliced RNAs generated by processing of the HIV-1 genomic RNA. Indicated are the common (open boxes) and alternative exons (closed boxes) used in the generation of the SS (4 kb) and MS (1.8 kb) viral RNAs. At the bottom is a list of the nomenclature used to refer to the exon composition of the individual RNAs generated for both the SS and MS classes of HIV-1 RNAs. (TIF) [file ppat.1003241.s001.tif]

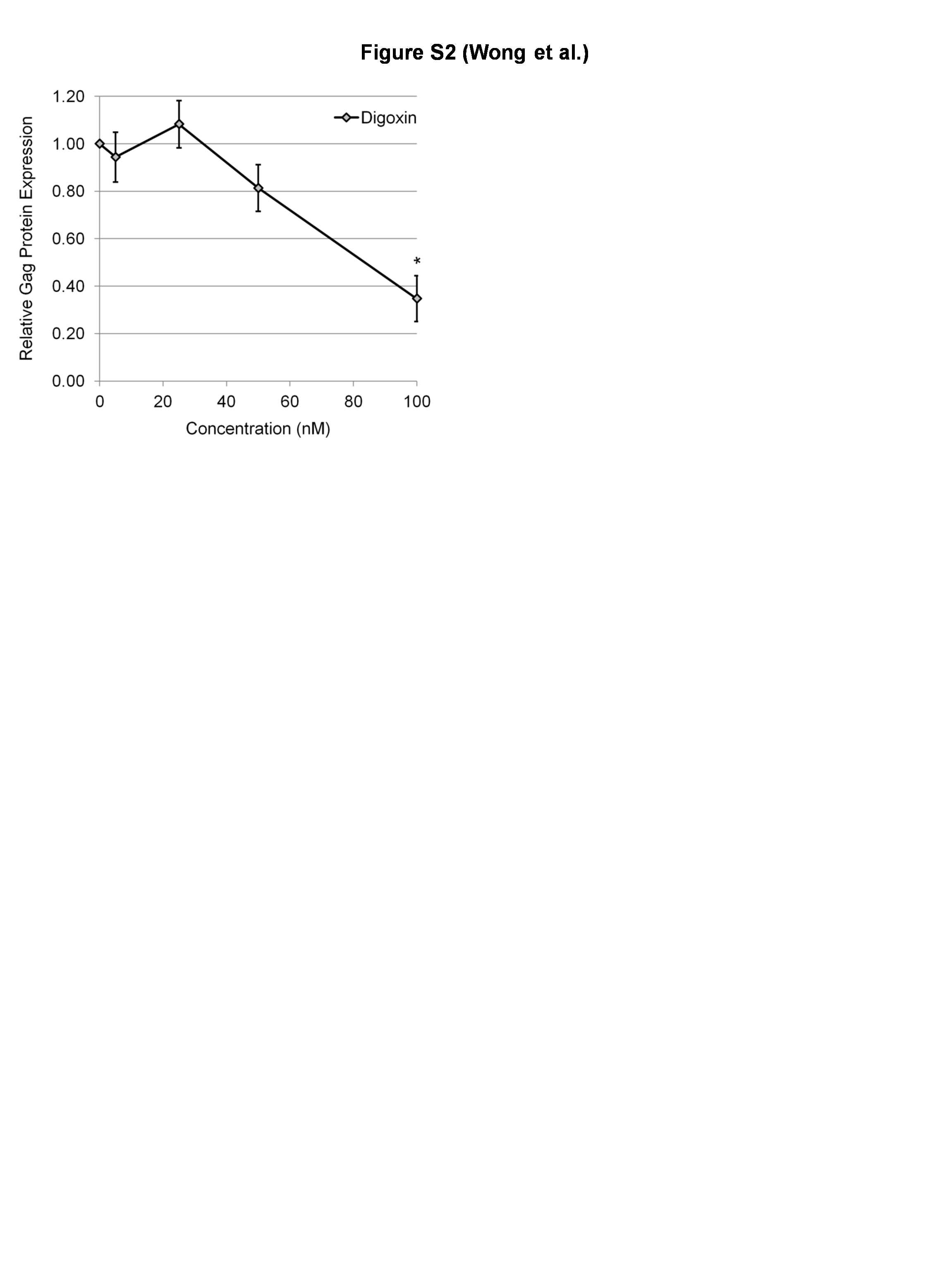

Supplement: Figure S2 — Digoxin inhibits HIV-1 (BaL) gene expression in PBMCs at day 3. Isolated PBMCs were infected with R5 HIV-1 BaL at a MOI of 10−2 in the presence of indicated doses of digoxin as described in Fig. 2. Equal concentrations of DMSO were present in each treatment. After 3 days, media was harvested for analysis of HIV-1 Gag protein expression by p24CA ELISA. Plotted data was averaged from ≥3 experiments, error bars are SEM, and asterisks indicate significant differences in digoxin treatment from DMSO control as detailed in “Materials & Methods”. (TIF) [file ppat.1003241.s002.tif]

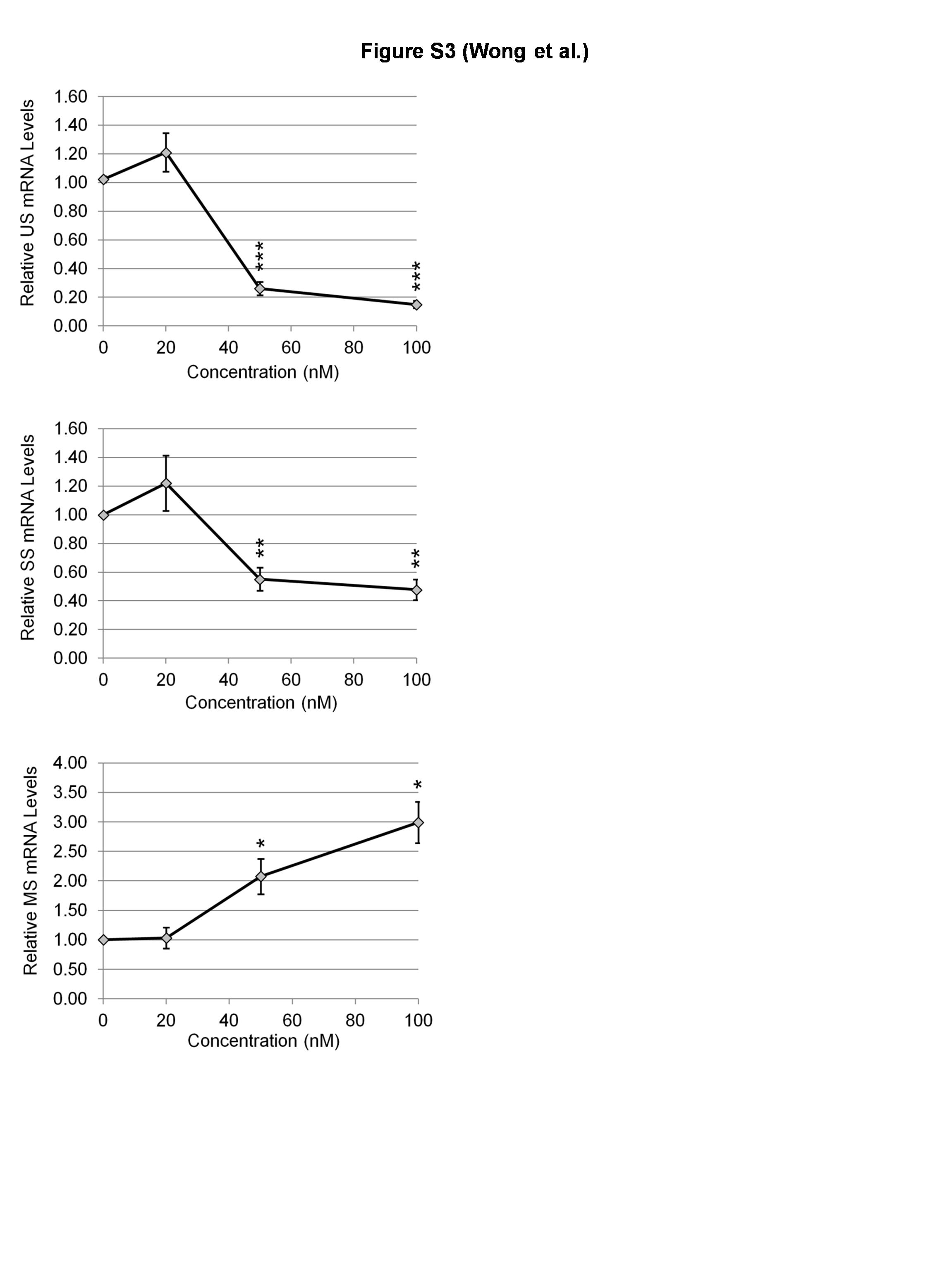

Supplement: Figure S3 — Digoxin alters HIV-1 RNA processing in a dose-dependent manner. The effect of increasing concentrations of digoxin on HIV-1 mRNA levels was assessed by qRT-PCR of cells treated with drug or DMSO as described in Fig. 1. Equal concentrations of DMSO were present in each treatment. Primers used in the amplification are described in Fig. 3 and “Materials & Methods”. The abundance of HIV-1 unspliced (US), singly spliced (SS), and multiply spliced (MS) mRNAs are shown relative to DMSO (+) control as indicated on the left. The housekeeping gene, β-actin, served as an internal loading control for the normalization of data. Data was averaged from ≥4 experiments, error bars are SEM, and asterisks indicate significant changes in treatments from DMSO (+) control as described in “Materials & Methods”. (TIF) [file ppat.1003241.s003.tif]

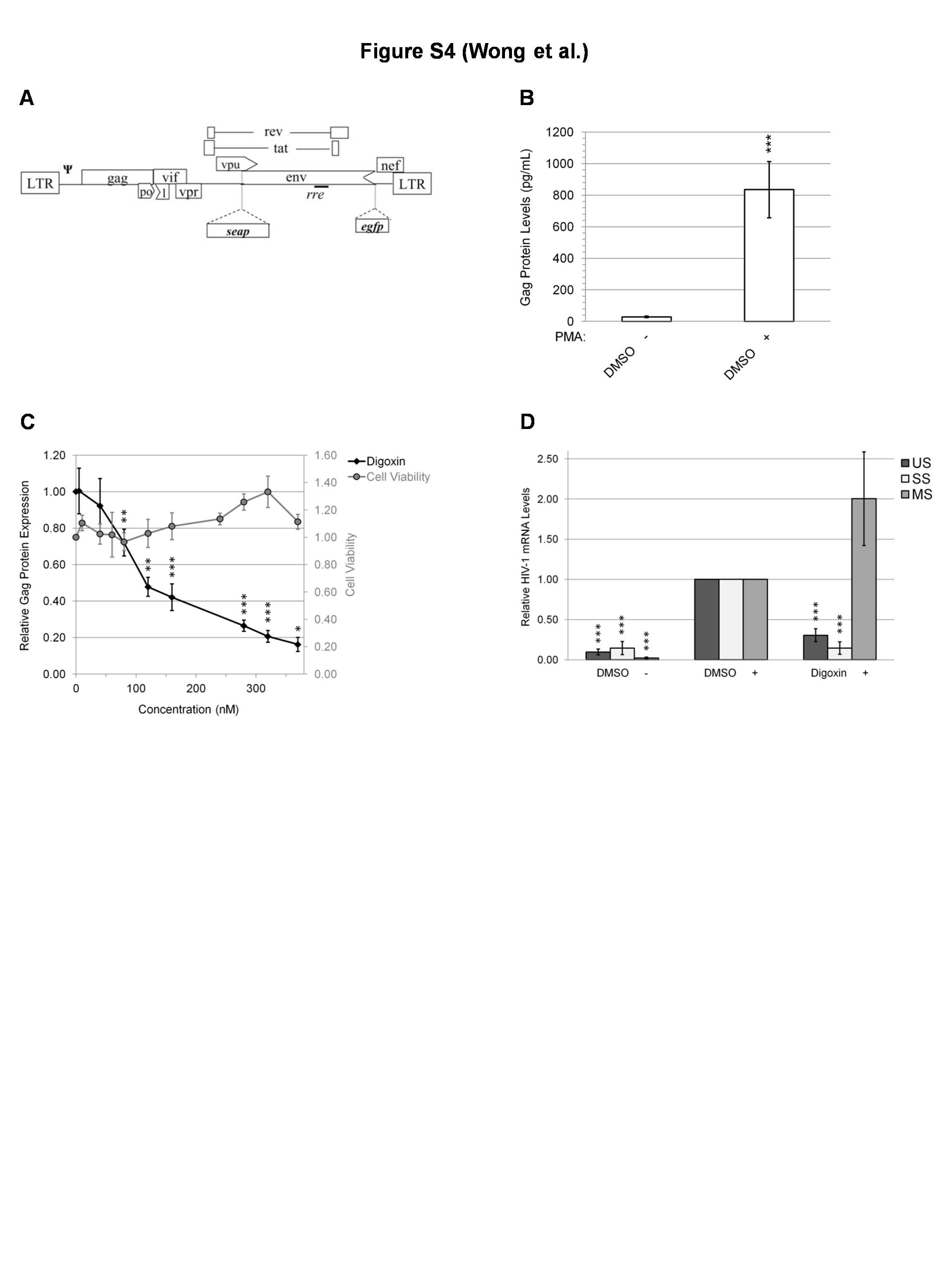

Supplement: Figure S4 — Effect of digoxin on HIV-1 expression and RNA processing in a chronic-infected human T cell line. (A) Schematic diagram of the HIV-1 provirus vector (NLE-S-G) used to generate the stably transduced SupT1 human T cell line, 24ST1NLESG [43]. (B) Induction of HIV-1 gene expression in 24ST1NLESG cells by PMA. Cells were incubated in the presence (+) or absence (−) of PMA (1.8 µM) and Gag expression was assayed 24 h after PMA addition by p24CA ELISA. Data was averaged from ≥11 experiments, error bars are SEM, and asterisks indicate significant change from DMSO (-PMA) as detailed in “Materials & Methods”. (C) Analysis of the effect of digoxin on HIV-1 gene expression in 24ST1NLESG cells. Cells (seeded at 1×10^6^ cells per mL in RPMI complete medium) were treated with indicated concentrations of digoxin for 4 h (prepared as described in “Materials and Methods” but in RPMI complete medium) and then HIV-1 gene expression was induced by addition of PMA. Equal concentrations of DMSO solvent were present in each treatment. After 24 h, cell media was harvested for quantitation of Gag expression by p24CA ELISA and cell viability was assessed in parallel by XTT assay (grey circles, secondary y-axis) as indicated. Shown is data averaged from ≥3–4 experiments, error bars are SEM, and asterisks indicate significant changes of treatment from DMSO (+) control as described in “Materials & Methods”. (D) Assessment of the effect of digoxin on HIV-1 mRNA accumulation. Cells were treated as outlined above, total RNA was extracted, and the abundance of US, SS, and MS forms of HIV-1 mRNA were quantitated by qRT-PCR as described in Fig. 3. Data was averaged from ≥5 experiments, error bars are SEM, and asterisks indicate significant changes in treatments from DMSO (+) controls as described in “Materials & Methods”. (TIF) [file ppat.1003241.s004.tif]

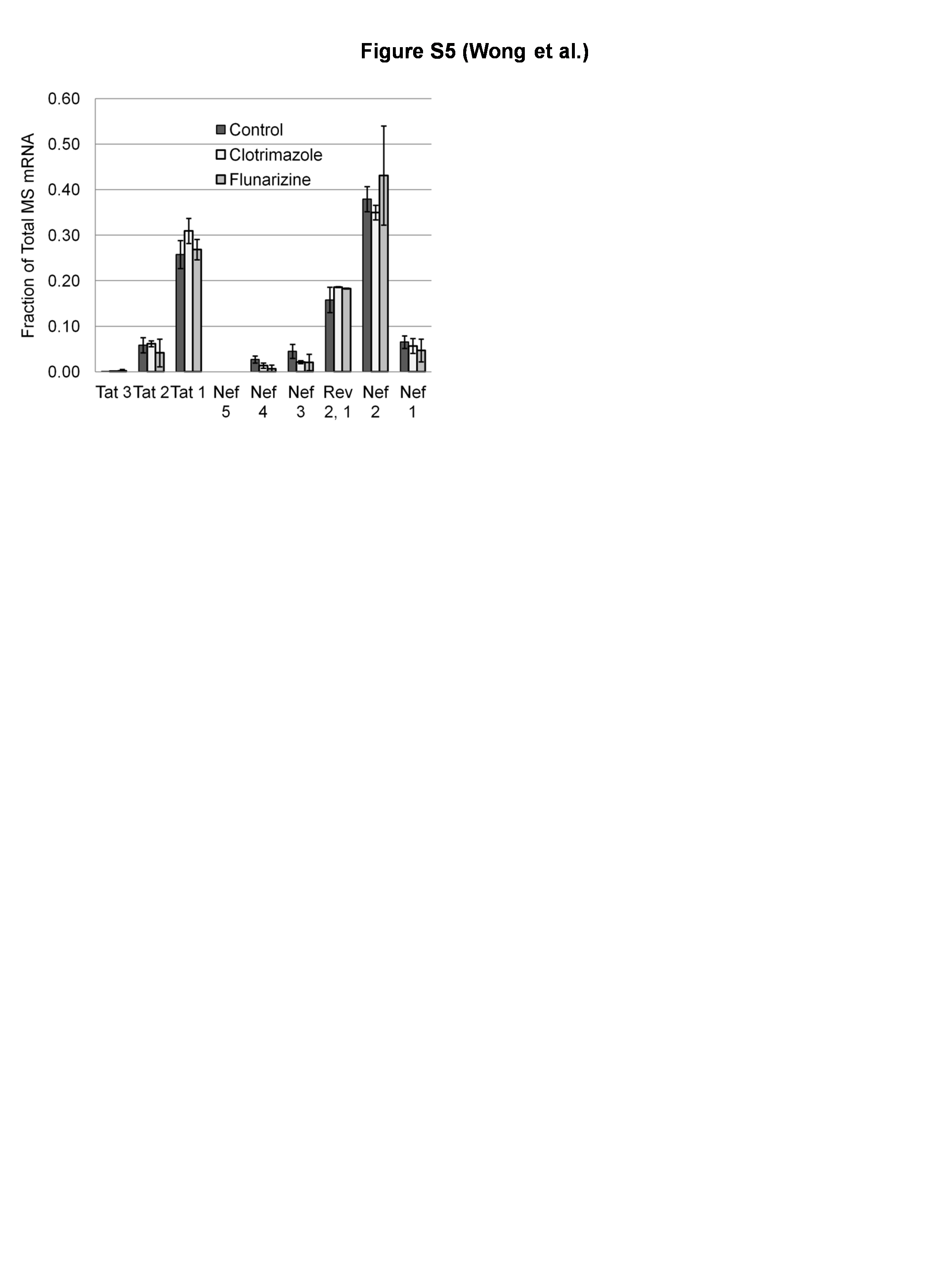

Supplement: Figure S5 — Clotrimazole and flunarizine do not affect HIV-1 pre-mRNA splice site usage. Effects of clotrimazole and flunarizine on HIV-1 splice site use were assayed by RT-PCR of the 2 kb, MS class of HIV-1 mRNA. Primers used to amplify HIV-1 MS mRNA species are described in Fig. 4 and “Materials & Methods”. This graph summarizes the effects of clotrimazole (white), flunarizine (gray), and control treatments (black) on the levels of each MS mRNA species (x-axis) relative to the total HIV-1 MS mRNA (y-axis), displayed as a fraction. Data was averaged from multiple experiments and error bars are SEM, and statistical significance calculated as described in “Materials & Methods”. (TIF) [file ppat.1003241.s005.tif]

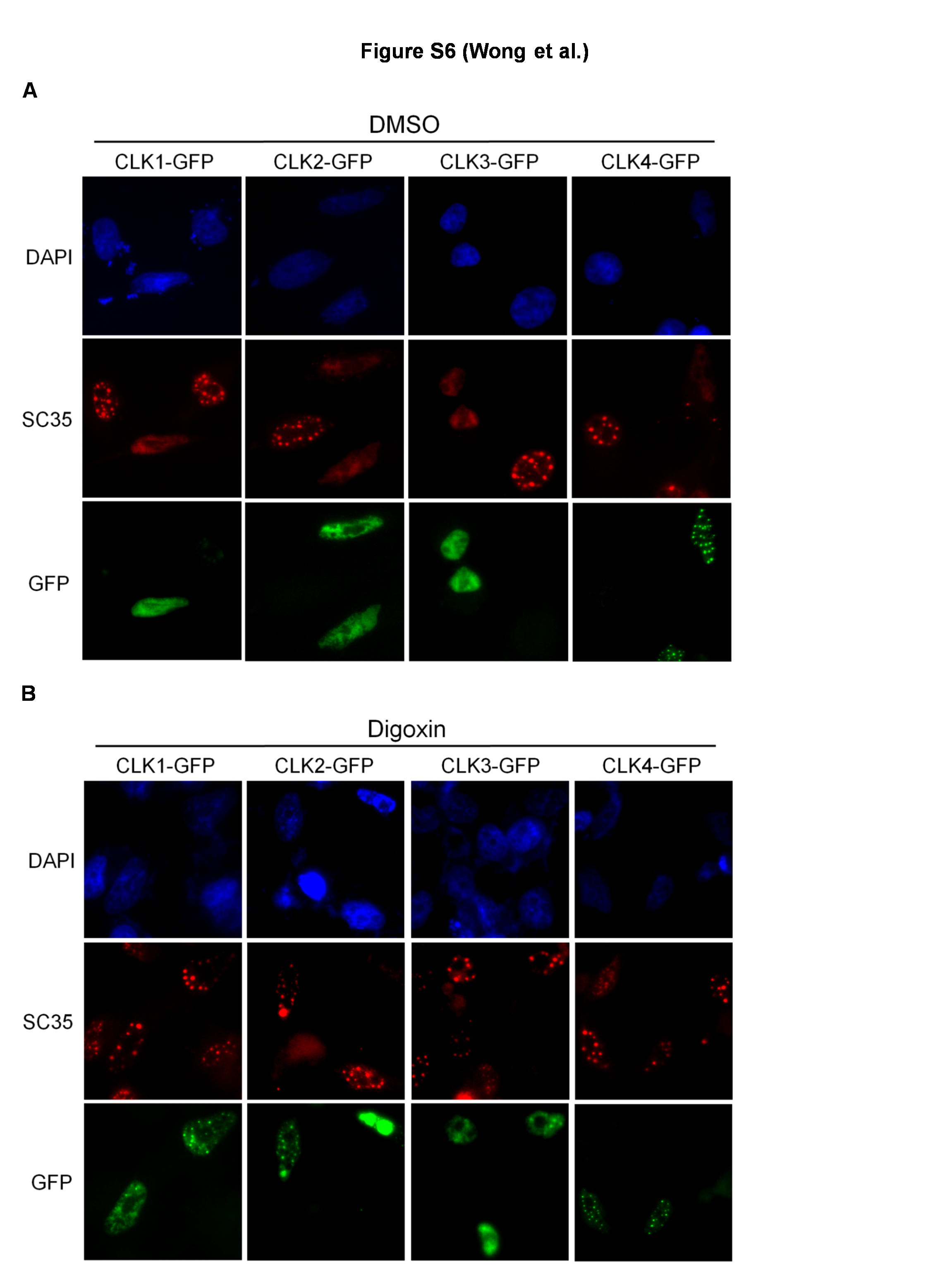

Supplement: Figure S6 — Digoxin alters the activity of CLK SR protein kinases. To assess the effect of digoxin on CLK kinase function, HeLa rtTA-HIV-ΔMls cells were transfected with vectors expressing GFP-tagged CLK1, CLK2, CLK3, or CLK4 as described in Fig. 6. Twenty-four hours post-transfection, cells were treated with (A) DMSO (control) or (B) 100 nM digoxin. After 24 h of treatment, cells were fixed, stained for SC35/SRSF2 nuclear speckles (Texas Red), and nuclei stained with DAPI. Images are representative of the localization patterns observed from ≥5 independent experiments. Magnification 630×. (TIF) [file ppat.1003241.s006.tif]
